# Tau Phosphorylation is Impacted by Rare *AKAP9* Mutations Associated with Alzheimer Disease in African Americans

**DOI:** 10.1007/s11481-018-9781-x

**Published:** 2018-03-07

**Authors:** Tsuneya Ikezu, Cidi Chen, Annina M. DeLeo, Ella Zeldich, M. Daniele Fallin, Nicholas M. Kanaan, Kathryn L. Lunetta, Carmela R. Abraham, Mark W. Logue, Lindsay A. Farrer

**Affiliations:** 1 Department of Pharmacology and Experimental Therapeutics, Boston University School of Medicine, Boston, MA 02118 USA; 2 Department of Neurology, Boston University School of Medicine, Boston, MA 02118 USA; 3 Department of Biochemistry, Boston University School of Medicine, Boston, MA 02118 USA; 4 Department of Mental Health, Johns Hopkins Bloomberg School of Public Health, Baltimore, MD 21205 USA; 5 Department of Translational Science and Molecular Medicine, Michigan State University, Grand Rapids, MI 49503 USA; 6 Department of Biostatistics, Boston University School of Public Health, Boston, MA 02118 USA; 7 Department of Medicine (Biomedical Genetics), Boston University School of Medicine, E200, 72 East Concord St., Boston, MA 02118 USA; 8 Department of Psychiatry, Boston University School of Medicine, Boston, MA 02118 USA; 9 The National Center for PTSD, Behavioral Science Division, VA Boston Healthcare System, Boston, MA 02130 USA; 10 Department of Ophthalmology, Boston University School of Medicine, Boston, MA 02118 USA; 11 Department of Epidemiology, Boston University School of Public Health, Boston, MA 02118 USA

**Keywords:** African American, Alzheimer disease, *AKAP9*, Amyloid-β peptide, APP, Tau, Rolipram

## Abstract

We studied the effect of two rare mutations (rs144662445 and rs149979685) in the A-kinase anchoring protein 9 (*AKAP9*) gene, previously associated with Alzheimer disease (AD) in African Americans (AA), on post-translational modifications of AD-related pathogenic molecules, amyloid precursor protein (APP) and microtubule-associated protein Tau using lymphoblastoid cell lines (LCLs) from 11 AA subjects with at least one *AKAP9* mutation and 17 AA subjects lacking these mutations. LCLs were transduced by viral vectors expressing causative AD mutations in APP or human full-length wild type Tau. Cell lysates were analyzed for total APP, Aβ_40_, and total and T181 phospho-Tau (pTau). *AKAP9* mutations had no effect on Aβ_40_/APP, but significantly increased pTau/Tau ratio in LCLs treated with phosphodiesterase-4 inhibitor rolipram, which activates protein kinase A. Proteomic analysis of Tau interactome revealed enrichment of RNA binding proteins and decrease of proteasomal molecules in rolipram-treated cells with *AKAP9* mutations. This study shows the impact of rare functional *AKAP9* mutations on Tau, a central mechanism of AD pathogenesis, in LCLs derived from AD and control subjects.

**Electronic supplementary material:**

The online version of this article (10.1007/s11481-018-9781-x) contains supplementary material, which is available to authorized users.

## Introduction

We previously identified by whole exome sequencing two rare specific variants in *AKAP9* (rs144662445 and rs149979685) in a sample of African American (AA) Alzheimer disease (AD) cases and controls (Logue et al. 2014). The odds of AD were increased 2.75-fold among individuals possessing one or both of these variants (*AKAP9*+). These SNPs were not present in more than 4000 sequenced individuals with European ancestry (The 1000 Genomes Project Consortium 2010), suggesting that this variant is unique to persons of African ancestry. *AKAP9* encodes A kinase-anchoring protein 9 (AKAP9), is ubiquitously expressed in the central nervous system and periphery, and is a scaffold protein that physically interacts with multiple protein kinase-related molecules, such as the regulatory subunit of protein kinase A (PPKAR2A) (Aranda et al. 2010), CDK5RAP2 (Wu et al. 2010), and GSK3B (Varjosalo et al. 2013). *AKAP9* has also been associated with long QT syndrome (Chen et al. 2007). However, the functional characterization of AA-specific variants has not been studied.

AKAP9 tethers protein kinase A (PKA) to subcellular loci via binding to the amino-terminal dimerization/docking (D/D) domain of PKA R subunits, and enhances its sensitivity to cAMP (Terrin et al. 2012). Amino acids 2327–2602 of the AKAP9 long isoform AKAP450 bind to PKA RII; a point mutation of amino acid 2566 of this isoform interferes with this interaction (Witczak et al. 1999). The AD-associated rs149979685 variant encodes a S3771 L mutation of the *AKAP9* Q99996–1 transcript and is predicted to have a functional consequence. The rs144662445 variant encodes an I2558M mutation of the *AKAP9* Q99996–1 transcript and is located within the PKA RII binding site, suggesting a loss-of-function mutation (Logue et al. 2014). It has been reported that PKA is biologically implicated in Tau phosphorylation, which is regulated by the intracellular level of and sensitivity to cyclic AMP (cAMP). PKA promotes the activity of glycogen synthase kinase-3β (GSK3B) and enhances Tau phosphorylation (Liu et al. 2006). The level of cAMP is regulated via its catalysis by phosphodiesterase 4A (PDE4A), which is known to interact with AKAP9 (Terrin et al. 2012). Tau phosphorylation by PKA, and a corresponding loss of PDE4A, confers risk for degeneration in aging association cortex (Carlyle et al. 2014). The role of PKA on amyloid-beta peptide (Aβ) generation from amyloid precursor protein (APP) is poorly understood. Because Tau and Aβ dysregulation are the hallmarks of AD pathogenesis, we hypothesized that AA AD-associated coding mutations in *AKAP9* might alter the activation status of PKA and increase phosphorylation of the Tau protein and Aβ production.

Non-brain cell sources have emerged as a powerful alternative for investigating gene expression differences ex vivo in neuropsychiatric disorders including schizophrenia, bipolar disorder and autism using peripheral blood leukocytes (Middleton et al. 2005; Tsuang et al. 2005; Hu et al. 2006) and lymphoblastoid cell lines (LCLs) (Kakiuchi et al. 2003; Vawter et al. 2004). LCLs have also been employed to study gene expression changes related to Aβ (Johnston et al. 1997; Hadar et al. 2016) and TDP-43 (Borroni et al. 2009). Taking into account that LCLs are easier to manipulate and culture than genetically engineered neurons or pluripotent stem cells, while faithfully recapitulating epigenomic signatures (Hadar et al. 2016) and providing material for multiple experiments, we tested the effect of AD-associated *AKAP9* missense mutations on Tau and Aβ using 28 LCLs from AD cases and controls with or without the rs144662445 and rs149979685 variants.

## Material and Methods

### Sample Selection

Eleven AA subjects included in our previous study (Logue et al. 2014) who had at least one of the *AKAP9* mutations for rs144662445 and rs149979685 (*AKAP9*+) and available lymphoblastoid cell lines were identified in the MIRAGE Study cell repository at Boston University and at the National Cell Repository for Alzheimer Disease (NCRAD) at Indiana University. All but one of these subjects had the mutation at both loci, while one subject had only the rs144662445 mutation. A comparison group of 17 AA subjects lacking the *AKAP9* mutations (*AKAP9*−) with corresponding cell lines was identified from the same sources. An attempt was made to match the two groups for age, sex, AD status, and *APOE* genotype (Table 1). *AKAP9*+ and *AKAP9*− subjects had similar proportions of females (91.1% vs. 94.1%) and mean ages (76.8 vs. 78.5 years). The *AKAP9*+ group had a higher proportion of AD subjects (63.6% vs. 47.1%) but fewer *APOE* ε4 carriers (45.5% vs. 58.8%).

**Table 1:** Subject characteristics by source of cell lines and presence or absence of *AKAP9* mutations

Source		*AKAP9*+	*AKAP9*−
MIRAGE Study	N	3	9
	Female (%)	3 (100%)	9 (100%)
	AD (%)	3 (100%)	4 (44.4%)
	*APOE* genotype 33	1 (33.3%)	2 (22.2%)
	34	1 (33.3%)	4 (44.4%)
	44	1 (33.3%)	3 (33.3%)
	Mean Age	82.3	78.9
NCRAD	N	8	8
	Female (%)	7 (87.5%)	7 (87.5%)
	AD (%)	4 (50%)	4 (50%)
	*APOE *genotype 23	2 (25%)	2 (25%)
	33	3 (37.5%)	3 (37.5%)
	34	3 (37.5%)	3 (37.5%)
	Mean Age	74.8	78.1
Total	N	11	17
	Female (%)	10 (91.1%)	16 (94.1%)
	AD (%)	7 (63.6%)	8 (47.1%)
	*APOE* genotype 23	2 (18.2%)	2 (11.8%)
	33	4 (36.4%)	5 (29.4%)
	34	4 (36.4%)	7 (41.2%)
	44	1 (9.1%)	3 (17.6%)
	Mean Age	76.8	78.5

### Construction of APPsw Lentiviral Plasmid and Lentivirus Stock Production

Full length human APP751 Swedish (APPsw) ^KM^670/671^NL^ mutant was PCR amplified (ClonAmp HiFi, Clontech) from APPsw in pcDNA3 vector (So et al. 2013) using the following primers: forward primer (5′ –TTACCTTCGAACCGCGGCTAGTTCTGCATCTGCTCAAAGAAC) and reverse primer (5′ – TAGCTCGAGTGCGGCCGCAACCCAG). Amplified product was subsequently cloned into the HFUW lentiviral vector (Chanoux et al. 2009) digested with EcoRI using In Fusion cloning kit (Clontech) following manufacturer’s protocol. The LV-APPsw plasmid was amplified in GCI-L3 *E. coli* cells (GeneCopoeia) to produce stable LV-APPsw plasmid. The plasmid was purified using ZymoPURE plasmid kit (Zymo). Lentivirus stocks were generated using the 293 T cell line co-transfected with LV-APPsw plasmid and two packaging plasmids (pMD2.G and psPAX2, Addgene #12259 and 12,260). Forty hours later, the supernatant was collected and filtered (0.45 mm) and stored frozen at −80 °C.

### Lymphoblastoid Cell Lines (LCLs) and Virus-Mediated Gene Transduction

LCLs were maintained in RPMI 1640 (Invitrogen) with 10% FBS (Atlanta Biologicals) with 1× Penicillin-Streptomycin (Invitrogen). Cells (2 million cells in T25 flask) were infected by either 1:3 dilution of LV-APPsw lentiviral stock solution or 4 μL of recombinant replication-incompetent adenovirus type 5 expressing human 2N4R microtubule-associated protein Tau (1–441) for overnight at 37 °C, followed by replacing tissue culture medium as described (Sato et al. 2006).

### Human Tau and pTau ELISA

After adenovirus infection, cells were subjected to 16 h incubation with 10 μM rolipram (CAS# 61413–54-5, Tocris, Minneapolis, MN), the PDE4 inhibitor that increases the intracellular cAMP level and activates AKAP-tethered PKA (McCahill et al. 2005), or control vehicle (0.5% dimethylsulfoxide in tissue culture medium). The cells were pelleted by centrifugation at 150 x g for 5 min at 4 °C, washed in ice-cold phosphate-buffered saline (PBS) and centrifuged, and the cell pellet was lysed in 5 volume of TENT buffer (20 mM Tris-Cl, pH 7.5, 2 mM ethylenediaminetetraacetic acid, 150 mM NaCl, 1% Triton X-100, 0.1 mM phenylmethylsulfonyl fluoride, 10 mM NaF, and 5 mM Na_3_VO_4_, all from Sigma-Aldrich, St. Louis, MO). The cell lysates were placed on ice and vortexed for 20 s every 5 min for 20 min, followed by centrifugation at 22,000 x g for 30 min to remove insoluble materials. The supernatant was transferred to a new tube and subjected to the quantification of total proteins by BCA assay (Cat# PI-23221, Pierce/Thermofisher Scientific, Inc.), total human Tau by ELISA (human Tau ELISA kit, Cat# KHB0041, Life Technologies), and pT181 pTau by ELISA (pTau [pT181] Human ELISA Kit, Cat# KHO0631, Life Technologies) as described (Asai et al. 2015). The pT181 pTau values were normalized by the total Tau value for each sample.

### APP and Aβ ELISA

After APPsw lentivirus infection for 48 h, the supernatants were collected for Aβ_40_ ELISA, and the cells were pelleted by centrifugation at 150 x g for 5 min at 4 °C, washed in ice-cold phosphate-buffered saline (PBS) and centrifuged, and the cell pellet was lysed in lysis buffer (10 mM Tris-HCl, pH 7.5, 50 mM NaCl, 50 mM NaF, 1% Triton X-100 and 10 mM EDTA) with protease cocktail inhibitor tablet (1 tablet per 50 mL, Roche). The conditioned medium and cell lysates were analyzed by SDS-PAGE and western blotting using APP antibody (6E10 mAb) in the cell lysate for total APP as described previously (Jun et al. 2014). Primary antibody for western blots was mouse monoclonal 6E10 (Covance, 1:1000) against amino acids 1–17 of Aβ that also recognizes human APP. ELISA was carried out using the human Aβ_40_ ELISA kit (Invitrogen) in accordance with manufacturer’s protocol with samples diluted 1:2 in diluent buffer.

### Statistical Analysis

The R statistical computing package (R Development Core Team 2008) was used to evaluate the association of *AKAP9* genotype with measures of Aβ and phosphorylated Tau. Specifically, the influence of *AKAP9* mutations (predictor) with normalized Aβ (Aβ/APP) and phosphorylated Tau (pTau/Tau) measures (outcomes) was analyzed in random-effects linear models including covariates for AD status and *APOE* genotype and a random effect term for sample to adjust for non-independence of repeated measures. The lmer function of lme4 R package (Bates et al. 2015) was used to fit the mixed models. Pairwise contrasts of differences between groups (e.g. *AKAP9*+, AD+ vs *AKAP9*+, AD−) were performed within the random-effects model framework using the multcomp R package (Hothorn et al. 2008) and results were illustrated using boxplots.

### Human Tau IP and Mass-Spectrometry

After adenovirus infection, a subset of cell lines were subjected to 16 h incubation with 20 μM rolipram (CAS# 61413–54-5, Tocris, Minneapolis, MN). Cells were then pelleted by centrifugation at 300 x *g* for 5 min at 4 °C. Supernatant was then removed and cells were lysed in 300 μL of TENT++ buffer (50 mM Tris-HCl pH 8, 2 mM ethylenediaminetetraacetic acid (EDTA), 150 nM NaCl, 1% Triton X-100, 0.5 mM phenylmethylsulfonyl fluoride (PMSF), 10 mM NaF, and 1 mM Na_3_VO_4_. All chemicals were from Sigma, except Na_3_VO_4,_ which was purchased from Alfa Aesar). Cells were vortexed for 1 min and then incubated on ice for 10 min, followed by centrifugation at 20,000 x *g* for 30 min at 4 °C to remove cell debris. The supernatant was then transferred to a fresh tube. BCA was used to quantify total protein amount. One mg of total protein was then processed for IP according to the instructions of the Pierce Direct IP column Kit with minor modifications (Cat #26148 Pierce/Thermofisher Scientific, Inc.). Columns used contained 10 μg of directly conjugated Tau13 antibody (provided by Binder/Kanaan laboratories) (Combs et al. 2016). Lysis buffer was replaced with TENT++. Samples were incubated in 45 μL NuPage LDS sample buffer (Cat# NP0007, Thermofisher Scientific, Inc.) for 20 min at 95 °C, and then eluted from the columns. The samples were then run for 1.5 cm in a 10% NuPage gel, and fixed for 30 min in 50% methanol, 40% miliQ water, and 10% acetic acid. The gels were then stained for 2 h in 50% methanol, 40% miliQ water, and 10% acetic acid and 0.25% *w*/*v* Coomassie brilliant blue R-250. Gels were destained overnight in 10% methanol, 7.5% acetic acid and 82.5% water. Following destaining, gels were washed twice for 10 min in miliQ water. Individual lanes were then extracted from the gel, stored in 10% acetic acid and sent to the mass spectrometry core at the University of Massachusetts Medical Center (Shrewsbury, MA) for processing.

### Proteomic Analysis

Resulting proteomic data were analyzed using the Scaffold 4 software. The 441-amino acid isoform of Tau was analyzed for the presence of post translational modifications (PTMs), which were visualized as proportion of modified reads out of total reads. These data were then analyzed for differences in PTMs of Tau protein according to *AKAP9* mutation or AD status using a paired t-test implemented in the Prism 6 software from Graphpad*.* The results of the t-tests were visualized in a paired differences graph where each point represents the proportion of modified reads in *AKAP9AKAP9*+ cells minus the proportion for the same site in *AKAP9*− cells or with AD+ lines minus the proportion for the same site in AD− cells. To understand functional differences in proteins co-precipitated with Tau, *AKAP9*+ or *AKAP9*− cell line proteins were filtered to retain only those proteins with at least two peptide spectra in all but one sample. Next, those protein expression levels were collapsed into a single number with the highest expression level being retained. Finally, the 400 most abundant proteins were selected in each condition and compared. Total abundance was determined by calculating normalized total spectra values using the iBAQ method within the Scaffold 4 software. The resulting 800 proteins were compared, and 51 emerged as unique to the top 400 of each condition (102 total). The 51 proteins from each condition were then analyzed by DAVID (Huang da et al. 2009a, b) to determine their ontologies. Only those ontologies with a *p*-value and False Discovery Rate (FDR, or *q*-value) less than 0.05 were retained. FDR was calculated by DAVID using the Benjamini method (Huang da et al. 2009a, b). Ontologies included are UP_KEYWORDS (UniProt Keywords), GOTERM_BP, CC, or MF_DIRECT (Gene ontology terms for Biological Processes, Cellular Component, or Molecular Function), and KEGG_Pathways (Kyoto Encyclopedia of Genes and Genomes).

## Results

### *AKAP9* Mutations Do Not Affect APP Processing or Aβ Production

**Fig. 1:**
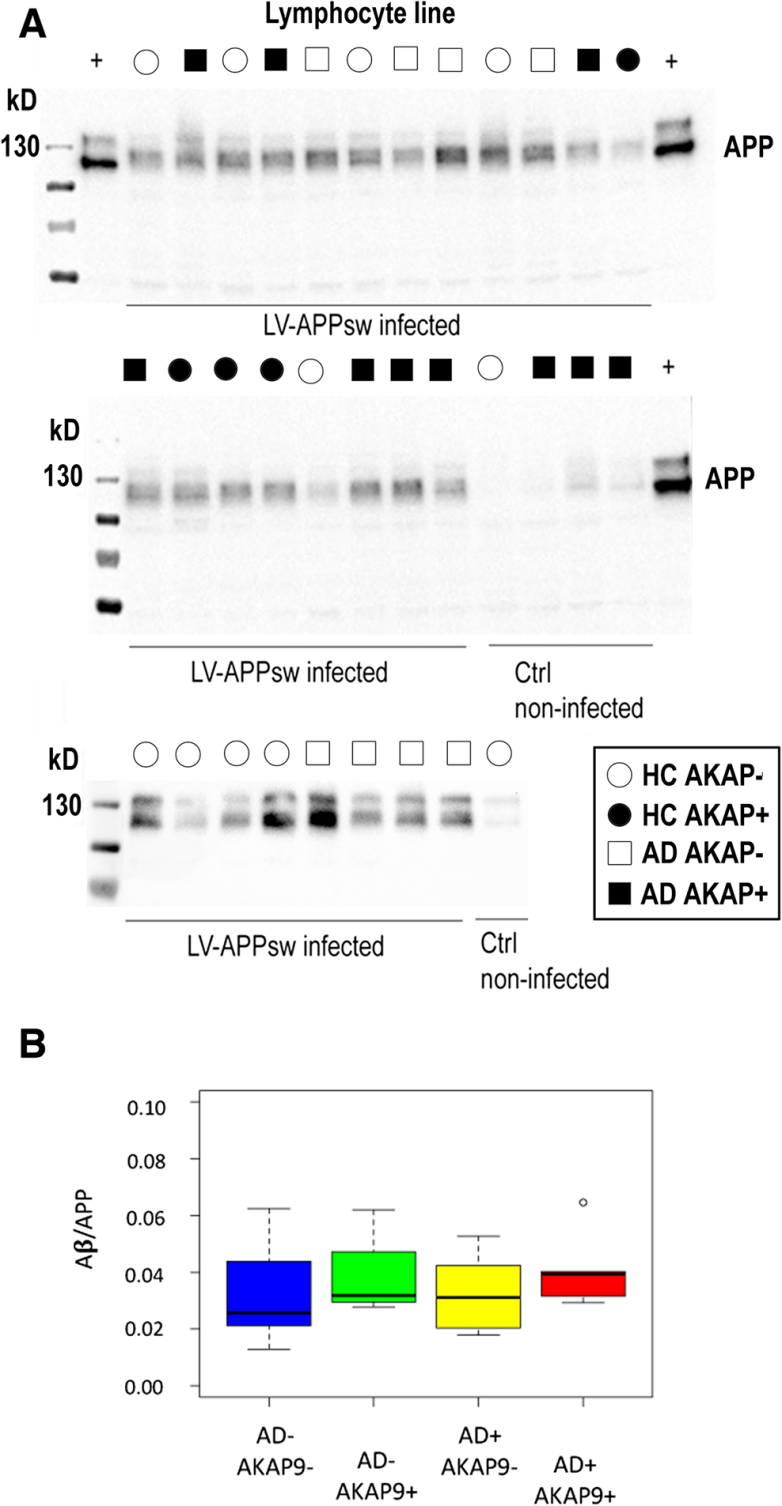
Measurement of Aβ_40_ production in lymphocytes infected with LV-APPsw. **a** Western Blot analysis of total APP in lentivirus-APPsw infected and control non-infected lymphocytes. A monoclonal antibody against APP (6E10) was used to detect endogenous and over expressed APP. The four groups tested were healthy controls (HC) *APAK9−*, HC *APAK9+*, AD *APAK9−*, AD *APAK9+*. APP transfected HEK cell lysate was used as a positive control for APP expression (+). **b** Mean Aβ_40_/APP ratio as a function of AD status and the presence of AD risk alleles for *AKAP9* variants rs144662445 and rs149979685. An Aβ_40_/APP ratio was used since each cell line was transfected separately, thus the levels of Aβ_40_ were relative to the levels of transfected APP

Our initial assessment of LCLs showed barely detectable secretion of endogenous human Aβ_40_ from these cell lines. Thus, we transduced recombinant human APP_751_ mutant carrying the ^KM^670/671^NL^ Swedish mutation into our LCLs. We examined the effect of *AKAP9* (rs144662445 and rs149979685) mutations on the production of APP and Aβ_40_ in LCLs. Figure 1a shows the results of western blots (WB) of 28 LCLs transfected transiently with the APP mutant. The Aβ_40_ released to the medium was analyzed by ELISA, and normalized to the total APP intensity as determined by densitometry from the WB in Fig. 1a. A boxplot of the Aβ_40_/APP ratio by *AKAP9* mutation and AD status is presented in Fig. 1b. The Aβ_40_/APP ratio was not significantly different as a function of AD status or *AKAP9* or APOE genotype (*p* > 0.10; Table 2).

**Table 2:** Association of Aβ/APP ratio with *AKAP9* mutations in a mixed model adjusting for AD status and *APOE* genotype

Parameter	Estimate	SE	Chisq	DF	p
*AKAP9 +* vs -	6.39E-04	5.09E-04	1.53	1	0.22
AD status	5.56E-05	4.88E-04	0.013	1	0.91
*APOE* 33 vs 23	1.37E-03	7.32E-04	5.76	3	0.12
34 vs 23	1.32E-03	7.18E-04			
44 vs 23	-2.78E-04	1.08E-03			

### *AKAP9* Mutations Increase Tau Phosphorylation in Rolipram-Treated Cells

**Fig. 2:**
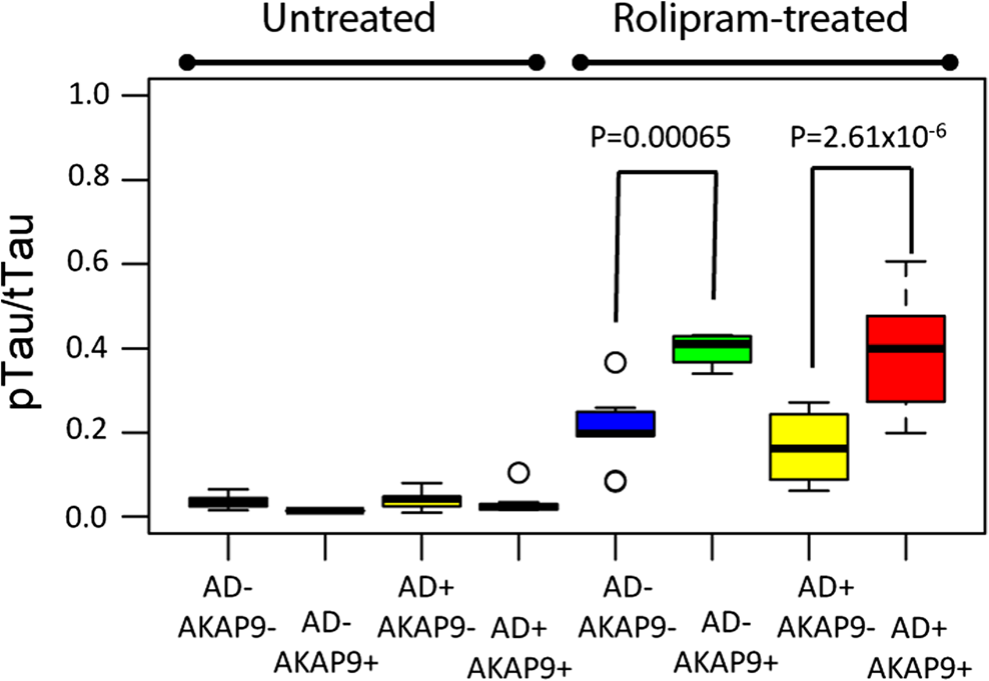
Mean pTau/Tau ratio as a function of AD status and the presence of AD risk alleles for *AKAP9* variants rs144662445 and rs149979685 in rolipram treated and untreated lymphoblastoid cell lines

We examined the effect of *AKAP9* mutations on Tau phosphorylation in our cell lines. AKAP9 tethers PKA and regulates its sensitivity to cAMP for this activation (Terrin et al. 2012). Since the basal cAMP was insufficient to monitor the effect of PKA activation, the cells were treated with roliplram. Rolipram is a PDE4 inhibitor that increases the intracellular cAMP level and activates AKAP-tethered PKA, and has previously been shown to increase Tau phosphorylation in conjunction with PKA (McCahill et al. 2005). Figure 2 presents a boxplot of the pTau/Tau ratio as a function of *AKAP9* and AD status for both the rolipram-treated and untreated samples. In the rolipram-untreated samples, none of the predictors are significant (all *p* > 0.09, Table 3a). In the mixed model of pTau/Tau for the treated samples (Table 3b), *AKAP9* mutation carrier status was the only significant predictor, with *AKAP9*+ subjects having higher pTau ratio than *AKAP9*− subjects (β = 0.21, *p* = 2.50 × 10^−6^). Post-hoc pairwise group comparisons indicate that pTau/Tau was significantly higher in *AKAP9*+ subjects within both AD cases (β = 0.22, *p* = 2.61 × 10^−6^) and cognitively normal controls (β = 0.19, *p* = 0.00065).

**Table 3:** Association of pTau/Tau Ratio with *AKAP9* mutations in a mixed model adjusting for AD status and *APOE* genotype in rolipram (A) untreated cell lines and (B) treated cell lines

Parameter	Estimate	SE	Chisq	DF	p
A.					
*AKAP9 +* vs -	−0.014	0.0083	2.73	1	0.098
AD status	0.0099	0.0081	1.46	1	0.23
*APOE* 33 vs 23	0.0021	0.012	0.71	3	0.87
34 vs 23	−0.0057	0.012			
44 vs 23	−0.00016	0.015			
B.					
*AKAP9 +* vs -	0.21	0.036	22.17	1	2.50E-06
AD status	−0.037	0.036	1.061	1	0.30
*APOE *33 vs 23	−0.033	0.055	1.66	3	0.65
34 vs 23	0.017	0.054			
44 vs 23	−0.02804	0.066			

### Tau IP-Proteomics

**Fig. 3:**
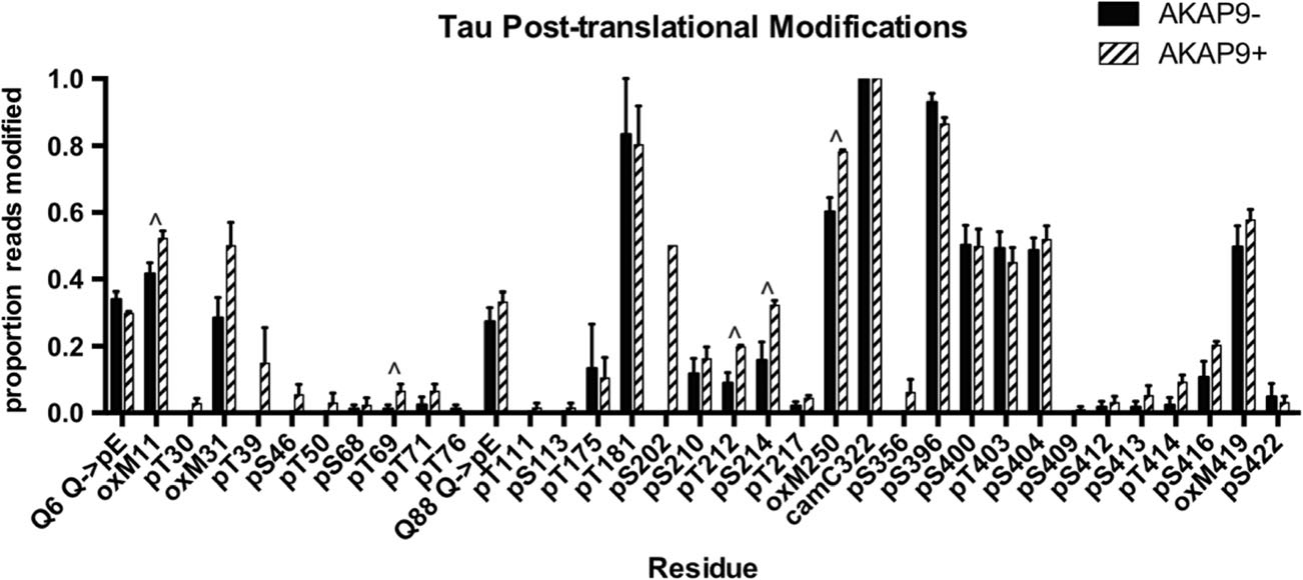
Proportions of post-translational modifications (PTMs) for reads of Tau protein derived from lymphocytes treated with rolipram as a function of AKAP9 mutation status. Plot shows proportion of reads with PTMs out of total as mean ± SEM of PTMs at each amino acid in the full-length 441 amino acid isoform of Tau. Residues with nominal *p*-values (less than 0.05) by t-test are indicated by a “^”

We then wanted to further investigate differences in post-translational modicfications of Tau and how this might alter the makeup of Tau-interacting proteins. Table 4 shows the proportion of observed post-translational modifications (PTMs) out of total reads of the protein Tau at individual amino acids as a function of AD and *AKAP9* mutation status following rolipram treatment for a subset of cell lines (*n* = 10). Differences in PTMs were then examined between *AKAP9* mutation carriers and non-carriers (Fig. 3). Although no PTMs were significant after adjusting for multiple testing, several PTMs, such as pT69, pT212, and pS214, were nominally significant at *p* < 0.05 (Fig. 3 and Table 4). There were no differences in Tau phosphorylation PTMs between cell lines derived from AD cases and controls (Fig. S1a).

**Table 4:**
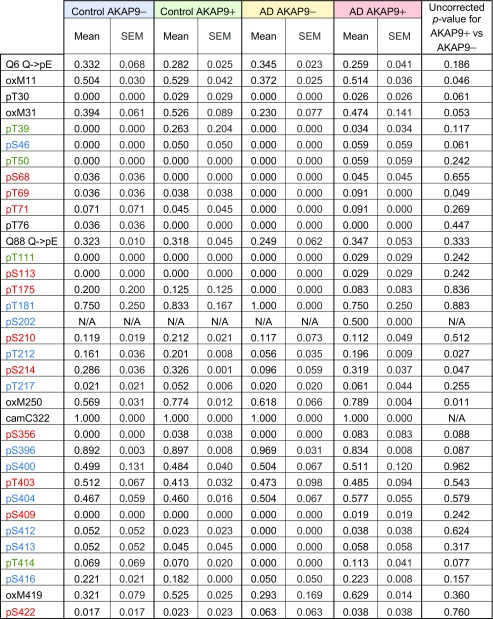
Post-translational modifications (PTMs) of Tau protein after rolipram treatment according to AD and *AKAP9* mutation status

Phosphorylation PTMs are colored in red by their known correlation with AD as previously reported (Luna-Munoz et al. 2007). Normal brain associated phosphorylation sites are labeled in green. Those found in both normal and AD brains are in blue. Putative phosphorylation sites not demonstrated at the time of the publication by Martin et al. (2011) are labeled in black

**Fig. 4:**
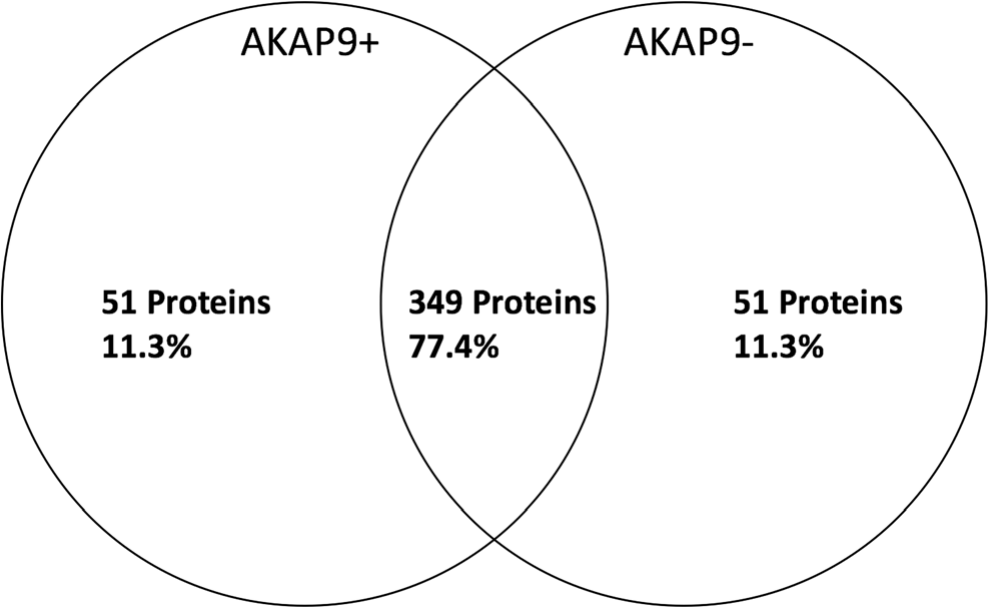
Venn diagram of top Tau-interacting 400 proteins for *AKAP9*+ and *AKAP9*− cell lines identified by Tau immunoprecipitation. All included proteins had at least two unique peptide spectra in all but one sample, and then were collapsed into single protein identities taking the highest expression value. The top three proteins for each category (*AKAP9*+ unique, left; *AKAP9*+and *AKA P9*− common, center; *AKAP9*− unique, right). The top three proteins for *AKAP9*+ cell lines are involved in RNA binding (ERH, Enhancer of Rudimentary Homolog; SNRPD1, Small Nuclear Ribonucleotide Protein D1 polypeptide; RPL11, Ribosomal Protein L11). *MAPT* (the gene encoding Tau) is common to the top three proteins of both *AKAP9*+ and *AKAP9*− cell lines (as well as IGKV2D-28, Immunoglobulin Kappa Variable 2D-28 and RPL39, Ribosomal Protein L39). The top three proteins unique to *AKAP9*− cell lines include a member of the proteasome family, PSMD8 (Proteasomal 26S Subunit, Non-ATPase 8). Other top unique proteins include IGKC, Immunoglobulin Kappa Constant and HLA-DQA2, Major Histocompatibility Complex, Class II DQ Beta 2

To help resolve patterns of Tau-interacting protein expression among *AKAP9* + and − cells, we selected the top 400 enriched proteins that had at least two unique peptide spectra in all but one sample by Tau-mediated immunoprecipitation. We observed that 51 of these proteins were expressed only in *AKAP9*+ cells and other 51 proteins were expressed only in *AKAP9*− cells (Fig. 4, Tables S1 and S2). Analysis of the function of these proteins by DAVID revealed that although *AKAP9* + and − cells contained distinct proteins, the proteins common to cells with and without *AKAP9* mutations were enriched for acetylation, extracellular exosomes, poly(A) RNA binding, cytoplasm and phosphoproteins (Table S3). *AKAP9*− cell lines included proteins involved in RNA binding, methylation, the spliceosome, the nucleus, and protein binding (Table S4). In contrast, *AKAP9*− cell lines included proteins involved in the proteasome, ubiquitination, prenylation, and cilia formation, as well as in NF-kappaB, Wnt, T-cell receptors, and TNF and Fc epsilon signaling pathways (Table S5). Interestingly, only the *AKAP9*− cell lines showed enrichment for ontologies related to the mitotic cell cycle, echoing earlier findings that the *AKAP9* mutations interfere with cell cycle progression (Logue et al. 2014).

Further ontological analysis revealed that acetylation, protein methylation, extracellular exosomes, and poly(A) RNA binding are common ontologies of Tau-interacting molecules in cell lines from both AD subjects and controls (Fig. S1b and Tables S6-S7). The most notable ontologies include phosphoproteins, mitochondria, and proteasomal molecules for proteins enriched only in the AD+ cell lines (Table S9). MHC class II receptors and other diseases such as lupus and tuberculosis were significant ontologies for proteins enriched only in cell lines from control subjects (Table S10).

Together, these findings suggest that the Tau-interactome can provide new insights about the effect of the *AKAP9* rs144662445 and rs149979685 mutations on AD risk and the biological functions of Tau.

## Discussion

This is one of only a few published functional genomic studies to assess the potential pathogenic effect of AD-associated genetic variants using primary lymphoblastoid cell lines (Johnston et al. 1997; Zou et al. 2013). To our knowledge, this is the first to study effects in cells from permutations of AD cases and cognitively normal controls with and without such variants. Our study leveraged phenotypic data previously assembled by the Alzheimer Disease Genetics Consortium for large-scale genetic studies in AAs (Reitz, et al. 2013) and genotypes for two AA-specific *AKAP9* mutations (I2558M and S3771 L) in the ADGC cohort (Logue et al. 2014). Although multiple reports demonstrated the presence of AKAP9 in human T cells (Herter et al. 2015; Robles-Valero et al. 2010; Skalhegg et al. 2005), we did not detect endogenous Tau and the APP level was barely detectable in the LCLs. We compensated for the low expression of these two AD-related proteins by employing recombinant virus-mediated expression of human full-length Tau or the familial AD-linked APP Swedish mutant. We observed generation of Aβ after expression of APPsw, demonstrating the existence of β/γ processing machinery of APP in human LCLs.

Our data show that these two amino acid substitutions in AKAP9 had no effect on the amyloidogenic APP processing pathway that generates Aβ. This finding also suggests that the *AKAP9* mutation(s) do not affect the interaction of AKAP9 with the regulatory subunit of PKA, nor do they alter the sensitivity of AKAP9 to cAMP for its activation.

The I2558M and S3771 L substitutions, however, significantly enhanced pTau levels in the presence of rolipram, the PDE4 inhibitor that increases the intracellular cAMP level. The increase in cAMP level triggered by rolipram mimics the NMDA-R dependent activation of excitatory pathways caused by cAMP-induced PKA activation seen early in the course of Alzheimer’s disease (van der Harg et al. 2017). Use of this drug allowed us to more readily understand the effect of the *AKAP9* mutations on Tau phosphorylation. The effect on phosphorylation was independent of clinically manifest AD, sex, or APOE genotype in this AA cohort. Our finding suggests that one or both of these mutations have a direct effect on the intracellular signaling leading to phosphorylation of Tau. Specifically, the T69, T212, and S214 residues show a nominally significant increase in phosphorylation in *AKAP9*+ cell lines when treated with rolipram. S214 is reported to be a direct phosphorylation site of PKA (Carlyle et al. 2014). Overall, the total phosphorylation of Tau is increased in *AKAP9*+ cell lines as compared to the *AKAP9*− cell lines. Since this biological phenotype is dependent on rolipram treatment, we assume that these *AKAP9* variants alter the sensitivity of PKA to cAMP, which results in the difference in the phosphorylation status of Tau. While many studies have shown beneficial effects of rolipram in AD model animals, high doses impaired prefrontal cortex function in aged monkeys, possibly owing to the different neurochemical needs of the prefrontal cortex in comparison with the hippocampus, and indicating that rolipram treatment may only benefit hippocampal functioning (Ramos et al. 2003, reviewed in Heckman et al. 2017). Furthermore, based on our results, rolipram treatment may not be beneficial in the long term, as we have found it to increase Tau phosphorylation.

Our findings suggest that altered *AKAP9* can lead to an increase in Tau phosphorylation, which is associated with AD. The mutations we studied, located in exons 36 and 51, respectively, are rare, and thus do not contribute substantially to AD risk in the general population. Moreover, because rs144662445 and rs149979685 *AKAP9* variants are co-segregating in nearly all subjects in this cohort, it is difficult to determine whether a particular mutation or the combination of both (I2558M or S3771 L) is responsible for the functional alteration. Only one *AKAP9*+ cell line had a single variant (I2558M). Although the treated pTau level in this cell line was in the range of scores observed in the other *AKAP9*+ cell lines, this is not enough information to perform a reliable statistical test. Vardarajan et al. recently observed association of AD risk with a distinct rare *AKAP9* missense mutation in exon 8 (R434W) in Caribbean Hispanic families with multiple members affected by AD (unpublished results). Recently, a genome-wide association study of AD-related endophenotypes in the ADNI study cohort subjects of European ancestry with mild cognitive impairment revealed significant association of several common SNPs (minor allele frequency, MAF = 0.45 and located 381 kb upstream of *AKAP9*) with pTau level (*p* < 6.9 × 10^−7^ for all SNPs) (Chung et al. 2018). In the same study, there was strong evidence for association of rs149454736 (MAF = 0.03), which is located between exons 45 and 46 of *AKAP9* and is only 1513 bp from rs149979865*,* with a brain MRI measure of hippocampal volume in AD subjects (*p* = 2.2 × 10^−7^). Further experiments are needed to determine whether these variants are functional and have an impact on Tau phosphorylation similar to the ones examined in this study.

Oxidation of the methionine residues of Tau was increased in rolipram-treated cells containing the *AKAP9* mutations (Fig. 3 and Table 4, residues M11, 31, 250 and 419; M11 and 250 nominally significant). A previous study reported that Tau aggregation is promoted by oxidation of the C322 residue present in the R3 region of the repeating microtubule binding domains found in all Tau isoforms (Martin et al. 2011; Schweers et al. 1995). However, the novel methionine PTMs identified in this study are located outside of the R3 region and therefore would not contribute to aggregation in the same manner as C322.

Protein ontology analysis revealed that there are key differences in the Tau interactome between the *AKAP9*+ and *AKAP9*− cell lines. The *AKAP9*+ cells were enriched in Tau-interacting proteins involved in protein methylation and the methylosome. Methylation of Tau protein has been reported in hAPP transgenic mice at locations corresponding to homologous residues of the human Tau 441 isoform (R126, R155, K163, K259, K311, K331, and R349) (Morris et al. 2015). Interestingly, the *AKAP9*+ cell lines were enriched for RNA binding proteins, including riboproteins and spliceosome proteins. Such proteins have been implicated in the formation of stress granules which accumulate in AD and co-localize with sites of Tau pathology (Vanderweyde et al. 2016). The *AKAP9*− cell lines are enriched for ontologies involved with Rab small GTP-binding proteins, proteasomal complex and cell cycle progression, a function previously ascribed to AKAP9 (Logue et al. 2014). These findings suggest that the *AKAP9* mutations may alter the Tau interactome and, consequently, alter its metabolism from Rab-mediated endosomal trafficking and proteasomal degradation and onto RNA binding protein-mediated aggregation in the cells.

There are limitations to our study worth noting. Although our previous study ruled out the possibility of other coding mutations in *AKAP9* or adjacent genes that are in linkage disequilibrium with the rs144662445 and rs149979685 as the functional AD-related variant at this locus (Logue et al. 2014), the true causal variant may be in a non-coding regulatory region. This idea is consistent with our recent observation that a SNP in the *KANSL1* gene promoter accounts for the AD association peak with *MAPT* (the gene encoding Tau) and co-regulates expression of alternatively spliced exon 3 in *MAPT* (Jun et al. 2016)*.* Another potential concern about these results is that *AKAP9* expression in lymphoblastoid cell lines may not hold true in neurons. Although further experiments in neuronal cells in culture or from brain tissue is a logical next step, the naturally occurring mutations studied here are rare (and thus unlikely to be found in brain repositories) and introducing them into cultured cells which lack important features of the genetic background may not mimic in vivo conditions. It was also somewhat surprising that we did not detect a difference in endogenous expression of APP in LCLs from AD cases compared to controls. This observation is consistent with a previous study which also did not find a difference in APP expression between non-familial AD cases and controls (Johnston et al. 1997). In addition, we did not observe endogenous expression of Tau in LCLs which, to our knowledge, has not been reported. It is possible that the lack of expression differences or expression per se of these proteins is due to their variable roles in LCLs and neurons. Alternatively, differential expression of Tau and APP in LCLs may be detectable only when adjusting for genetic influences (e.g., *AKAP9* mutations) that directly impact their expression.

In conclusion, this study showed that lymphoblastoid cell lines derived from persons possessing the AKAP9 I2558M and/or S3771 L variants exhibit significant alteration in phosphorylation of recombinant Tau protein after virus-mediated transient expression of Tau and chemical inhibition of PDE4 by rolipram. This alteration in Tau phosphorylation was independent of AD status and *APOE* genotype. The presence of *AKAP9* mutations altered the composition of Tau-interacting proteins, increasing interaction of RNA and protein binding molecules, while proteasomal proteins were enriched in cell lines lacking these mutations. The approach of using human-derived cell lines for ex-vivo evaluation of pathogenic effects of AD-associated polymorphisms could be widely applicable to other diseases as a new modality of functional genomics, in addition to 3-dimensional tissue culture models of human neuronal cells (Choi et al. 2014; Kim et al. 2015; Shi et al. 2012).

## Electronic supplementary material

ESM 1 (DOCX 291 kb)

ESM 2 (DOCX 114 kb)
