# Supplementary material for: Tau Phosphorylation is Impacted by Rare AKAP9 Mutations Associated with Alzheimer Disease in African Americans
Source: J Neuroimmune Pharmacol. 2018 Mar 7;13(2):254–64. doi: 10.1007/s11481-018-9781-x (PMC5928172; doi:10.1007/s11481-018-9781-x)

**Figure S1:** *PTMs of tau protein derived from lymphocytes treated with rolipram as a function of AD status.* (A) Plot shows proportion of reads with PTMs out of total as mean ± SEM of PTMs at each amino acid in the full-length 441 amino acid isoform of tau. Residues with nominal p-values < 0.05 by t-test are indicated by a “^”. (B) Plot showing differences in each PTM site for tau (p = 0.9 by paired t-test). (C) Venn diagram of top 400 proteins in AD+ vs AD- conditions. Top three proteins for AD+ condition: SLIRP, SRA Stem-Loop Interacting RNA-Binding Protein; FKBP1A, FK506 Binding Protein 1A; PSMD8, Proteosome 26S subunit, Non-ATPase 8. Common top three proteins: IGKV2D-28, Immunoglobullin Kappa Variable 2D-28; MAPT, Microtubule Associated Protein Tau; RPL39, Ribosomal Protein L39. Top three proteins for the AD- condition: ERH, Enhancer of Rudimentary Homolog; H2AFZ, H2A Histone Family Member Z; CALML5, Calmodulin Like 5.


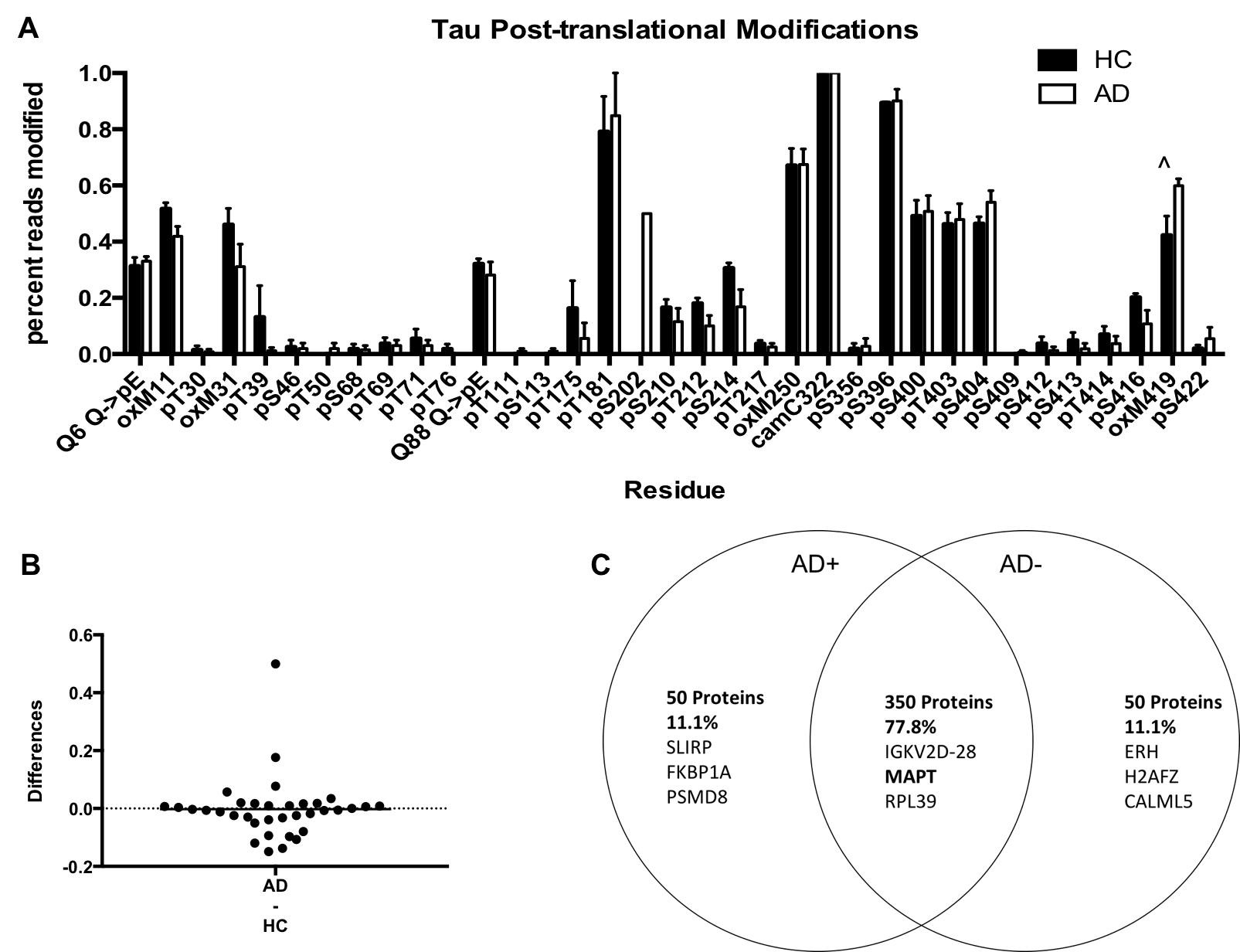

Supplement: Supplementary file 1 — (DOCX 291 kb) [file 11481_2018_9781_MOESM1_ESM.docx]
