# Supplementary material for: Tau Phosphorylation is Impacted by Rare AKAP9 Mutations Associated with Alzheimer Disease in African Americans
Source: J Neuroimmune Pharmacol. 2018 Mar 7;13(2):254–64. doi: 10.1007/s11481-018-9781-x (PMC5928172; doi:10.1007/s11481-018-9781-x)
Supplement: Supplementary file 2 — (DOCX 114 kb) [file 11481_2018_9781_MOESM2_ESM.docx]

Table of Contents

[**Table S1.** Tau interactome enriched in *AKAP9*+ group 2](#_Toc507068256)

[**Table S2.** Tau interactome enriched in *AKAP9*− group 7](#_Toc507068257)

[**Table S4.** Ontologies of Tau interactome enriched in *AKAP9*+ group 15](#_Toc507068258)

[**Table S5.** Ontologies of Tau interactome enriched in *AKAP9*− group 17](#_Toc507068259)

[**Table S6.** Tau interactome unique to AD+ group 20](#_Toc507068260)

[**Table S7.** Tau interactome unique to AD− group 27](#_Toc507068261)

[**Table S9.** Ontologies of Tau interactome enriched in AD+ group 33](#_Toc507068262)

[**Table S10.** Ontologies of Tau interactome enriched in AD− group 35](#_Toc507068263)

# **Table S1.** Tau interactome enriched in *AKAP9*+ group

| **Gene Symbol** | **UniProtKB** | **Gene Name** | **Function (GeneCards)** |
| --- | --- | --- | --- |
| ERH | P84090 | Enhancer of Rudimentary homolog (Drosophila) | No information |
| SNRPD1 | P62314 | Small nuclear ribonucleoprotein Sm D1 | Core component of the spliceosomal U1, U2, U4 and U5 small nuclear ribonucleoproteins (snRNPs). |
| RPL11 | P62913 | Ribosomal Protein L11 | Belongs to the L5P family of ribosomal proteins. Located in the cytoplasm. Required for rRNA maturation & formation of the 60S ribosomal subunits. Promotes nucleolar location of PML. |
| LPXN | O60711 | Leupaxin | Transcriptional coactivator for androgen receptor (AR) and serum response factor (SRF). Contributes to the regulation of cell adhesion, spreading and cell migration. Acts as a negative regulator in integrin-mediated cell adhesion events. Suppresses the integrin-induced tyrosine phosphorylation of paxillin. Negatively regulates B-cell antigen receptor (BCR) signaling. |
| S100A7 | P31151 | S100 Calcium Binding Protein A7 | Member of the S100 family of proteins containing 2 EF-hand calcium-binding motifs. Localized in the cytoplasm and/or nucleus of a wide range of cells. Involved in the regulation of a number of cellular processes such as cell cycle progression & differentiation. Overexpressed in hyperproliferative skin diseases. Exhibits antimicrobial activities against bacteria. Induces immunomodulatory activities. |
| SNRPGP15 | A8MWD9 | Small Nuclear Ribonucleoprotein Polypeptide G Pseudogene 15 | A pseudogene, affiliated with the antisense RNA class. |
| FKBP1A | P62942 | FK506 Binding Protein 1A | Cis-trans prolyl isomerase that binds the immunosuppressants FK506 and rapamycin. Interacts with several intracellular signal transduction proteins including type I TGF-beta receptor, with multiple intracellular calcium release channels & coordinates multi-protein complex formation of the tetrameric skeletal muscle ryanodine receptor. Keeps in an inactive conformation TGFBR1, the TGF-beta type I serine/threonine kinase receptor, preventing TGF-beta receptor activation in absence of ligand. Recruits SMAD7 to ACVR1B preventing the association of SMAD2 & SMAD3 with the activin receptor complex, blocking the activin signal. PPIases accelerate the folding of proteins. Catalyzes the cis-trans isomerization of proline imidic peptide bonds in oligopeptides. |
| HLA-DPB1 | P04440 | HLA class II histocompatibility antigen, DP beta 1 chain | Binds peptides derived from antigens that access the endocytic route of antigen presenting cells (APC) and presents them on the cell surface for recognition by the CD4 T-cells. |
| HSPB1 | P04792 | Heat shock protein beta-1 | Small heat shock protein which functions as a molecular chaperone. Plays a role in stress resistance and actin organization. |
| KRT33B | Q14525 | Keratin, type I cuticular Ha3-II | As a type I hair keratin, the encoded protein is an acidic protein which heterodimerizes with type II keratins to form hair and nails. |
| RUVBL1 | Q9Y265 | RuvB Like AAA ATPase 1 | Possesses single-stranded DNA-stimulated ATPase and ATP-dependent DNA helicase (3' to 5') activity. Component of the NuA4 histone acetyltransferase complex, involved in transcriptional activation of select genes by acetylation of nucleosomal histones H4 and H2A. Plays an essential role in oncogenic transformation by MYC. Modulates transcriptional activation by the LEF1/TCF1-CTNNB1 complex. Essential for cell proliferation. |
| RPL35A | P18077 | Ribosomal protein L35a | Belongs to the L35AE family of ribosomal proteins. Located in the cytoplasm. The rat protein binds to both initiator and elongator tRNAs, and thus, it is located at the P site, or P & A sites, of the ribosome. Required for the proliferation & viability of hematopoietic cells. Plays a role in 60S ribosomal subunit formation. |
| CSTA | P01040 | Cystatin-A | Intracellular thiol proteinase inhibitor. Has an important role in desmosome-mediated cell-cell adhesion in the lower levels of the epidermis. Encodes a stefin that functions as a cysteine protease inhibitor, forming tight complexes with papain and the cathepsins B, H, and L. One of the precursor proteins of cornified cell envelope in keratinocytes and plays a role in epidermal development and maintenance. |
| USMG5 | Q96IX5 | Up-regulated during skeletal muscle growth protein 5 | Plays a critical role in maintaining the ATP synthase population in mitochondria. |
| RHOG | P84095 | Rho-related GTP-binding protein RhoG | Required for the formation of membrane ruffles during macropinocytosis. Plays a role in cell migration and is required for the formation of cup-like structures during trans-endothelial migration of leukocytes. |
| PCMT1 | P22061 | Protein-L-isoaspartate(D-aspartate) O-methyltransferase | Catalyzes the methyl esterification of L-isoaspartyl and D-aspartyl residues in peptides and proteins that result from spontaneous decomposition of normal L-aspartyl and L-asparaginyl residues. Plays a role in the repair and/or degradation of damaged proteins. |
| IL4I1 | Q96RQ9 | Interleukin 4 Induced 1, L-amino-acid oxidase | Lysosomal L-amino-acid oxidase with highest specific activity with phenylalanine. Encodes a protein with limited similarity to L-amino acid oxidase which contains the conserved amino acids thought to be involved in catalysis and binding of flavin adenine dinucleotide cofactor. |
| CD74 | P04233 | HLA class II histocompatibility antigen gamma chain | Plays a critical role in MHC class II antigen processing by stabilizing peptide-free class II alpha/beta heterodimers in a complex soon after their synthesis. Directs transport of the complex from the endoplasmic reticulum to the endosomal/lysosomal system where the antigen processing and binding of antigenic peptides to MHC class II takes place. Serves as cell surface receptor for the cytokine MIF. |
| SSR4 | P51571 | Signal Sequence Receptor Subunit 4, Translocon-associated protein subunit delta | Encodes the delta subunit of the translocon-associated protein complex which is involved in translocating proteins across the endoplasmic reticulum membrane. |
| RBMX | P38159 | RNA-binding motif protein, X chromosome | This gene belongs to the RBMY gene family. This gene, an active X chromosome homolog of the Y chromosome RBMY gene, is widely expressed whereas the RBMY gene evolved a male-specific function in spermatogenesis. Involved in the cytoplasmic TNFR1 trafficking pathways; promotes the IL-1-beta-mediated inducible proteolytic cleavage of TNFR1 ectodomains and the release of TNFR1 exosome-like vesicles to the extracellular compartment. |
| VDAC2 | P45880 | Voltage-dependent anion-selective channel protein 2 | Forms a channel through the mitochondrial outer membrane that allows diffusion of small hydrophilic molecules. The channel adopts an open conformation at low or zero membrane potential and a closed conformation at potentials above 30-40 mV. The open state has a weak anion selectivity whereas the closed state is cation-selective. |
| PRMT1 | Q99873 | Protein arginine methyltransferase 1 | Arginine methyltransferase that methylates (mono and asymmetric dimethylation) the guanidino nitrogens of arginyl residues. Main enzyme that mediates monomethylation and asymmetric dimethylation of histone H4 'Arg-4', a specific tag for epigenetic transcriptional activation. With dimethylated PIAS1, represses STAT1 transcriptional activity, in the late phase of interferon gamma signaling. |
| RAB5B | P61020 | RAB5B, Member RAS Oncogene Family | Involved in protein transport. |
| RAB11B | Q15907 | RAB11B, Member RAS Oncogene Family | Required for melanosome transport and release from melanocytes. Also regulates V-ATPase intracellular transport in response to extracellular acidosis. |
| DNAJB11 | Q9UBS4 | DnaJ homolog subfamily B member 11 | Serves as a co-chaperone for HSPA5. Binds directly to both unfolded proteins that are substrates for ERAD and nascent unfolded peptide chains but dissociates from the HSPA5-unfolded protein complex before folding is completed. Stimulates HSPA5 ATPase activity. |
| TAF15 | Q92804 | TATA-Box Binding Protein Associated Factor 15 | Encodes a member of the TET family of RNA-binding proteins. Plays a role in RNA polymerase II gene transcription as a component of a distinct subset of multi-subunit transcription initiation factor TFIID complexes. |
| ARG1 | P05089 | Arginase 1 | Arginase is involved in step 1 of the subpathway that synthesizes L-ornithine and urea from L-arginine. The type I isoform encoded by this gene is a cytosolic enzyme and is expressed predominantly in the liver as a component of the urea cycle. |
| C1QBP | Q07021 | Complement component 1 Q binding protein (mitochondrial) | Putative receptor for C1q; specifically binds to the globular "heads" of C1q thus inhibiting C1. Involved in regulation of RNA splicing by inhibiting the RNA-binding capacity of SRSF1 and its phosphorylation. Required for the nuclear translocation of splicing factor U2AF1L4. Involved in regulation of CDKN2A- and HRK-mediated apoptosis. Stabilizes mitochondrial CDKN2A isoform smARF. |
| KIAA0391 | O15091 | Mitochondrial ribonuclease P protein 3 | Functions in mitochondrial tRNA maturation. Part of mitochondrial ribonuclease P, an enzyme composed of MRPP1/TRMT10C, MRPP2/HSD17B10 and MRPP3/KIAA0391, which cleaves tRNA molecules in their 5'-ends. |
| HNRNPM | P52272 | Heterogeneous nuclear ribonucleoprotein M | Pre-mRNA binding protein. Involved in splicing. Acts as a receptor for carcinoembryonic antigen in Kupffer cells. |
| RBBP4 | Q09028 | RB Binding Protein 4, Chromatin Remodeling Factor | A ubiquitously expressed nuclear protein belonging to a highly conserved subfamily of WD-repeat proteins. Component of several complexes which regulate chromatin metabolism, including chromatin assembly factor 1 (CAF-1) complex, core histone deacetylase (HDAC) complex, nucleosome remodeling & histone deacetylase complex (the NuRD complex), PRC2/EED-EZH2 complex, & the NURF (nucleosome remodeling factor) complex. |
| DDX3X | O00571 | DEAD-Box Helicase 3, X-Linked | Multifunctional ATP-dependent RNA helicase. ATPase activity can be stimulated by various ribo- and deoxynucleic acids indicative for a relaxed substrate specificity. Involved in several steps of gene expression, such as transcription, mRNA maturation, mRNA export, translation, transcriptional regulation, translation initiation. Associated with the E-cadherin promoter and can down-regulate transcription from the promoter. Promotes translation of a subset of viral and cellular mRNAs carrying a 5'proximal stem-loop structure in their 5'UTRs and cooperates with the eIF4F complex. Acts as an antiapoptotic protein through association with GSK3A/B and BIRC2 in an apoptosis antagonizing signaling complex; activation of death receptors promotes caspase-dependent cleavage of BIRC2 and DDX3X and relieves the inhibition. |
| RUVBL2 | Q9Y230 | RuvB-like 2 | Possesses single-stranded DNA-stimulated ATPase and ATP-dependent DNA helicase (5' to 3') activity. Component of the NuA4 histone acetyltransferase complex, involved in transcriptional activation of select genes principally by acetylation of nucleosomal histones H4 and H2A. Plays an essential role in oncogenic transformation by MYC. Modulates transcriptional activation by the LEF1/TCF1-CTNNB1 complex. Involved in the endoplasmic reticulum (ER)-associated degradation (ERAD) pathway where it negatively regulates expression of ER stress response genes. |
| SYNGR2 | O43760 | Synaptogyrin-2 | Encodes an integral membrane protein containing four transmembrane regions and a C-terminal cytoplasmic tail that is tyrosine phosphorylated. |
| SRSF1 | Q07955 | Serine and arginine-rich splicing factor 1 | Plays a role in preventing exon skipping, ensuring the accuracy of splicing and regulating alternative splicing. Interacts with other spliceosomal components to form a bridge between the 5'- and 3'-splice site binding components, U1 snRNP and U2AF. Can stimulate binding of U1 snRNP to a 5'-splice site-containing pre-mRNA. Binds to purine-rich RNA sequences. |
| TPM4 | P67936 | Tropomyosin 4 | Binds to actin filaments in muscle and non-muscle cells. Plays a central role, with the troponin complex, in the calcium dependent regulation of striated muscle contraction. In non-muscle cells is implicated in stabilizing cytoskeleton actin filaments. Binds calcium. |
| C14orf166 | Q9Y224 | UPF0568 protein C14orf166 | RNA-binding protein involved in modulation of mRNA transcription by Polymerase II. Component of the tRNA-splicing ligase complex and is required for tRNA ligation. |
| DHPS | P49366 | Deoxyhypusine synthase | Required for the formation of hypusine, a unique amino acid formed by the posttranslational modification of only one protein, eukaryotic translation initiation factor 5A. |
| LSP1 | P33241 | Lymphocyte-specific protein 1 | An intracellular F-actin binding protein. Expressed in lymphocytes, neutrophils, macrophages, & endothelium. |
| EWSR1 | Q01844 | EWS RNA-binding protein 1 | A multifunctional protein involved in gene expression, cell signaling, RNA processing & transport. Includes an N-terminal transcriptional activation domain & C-terminal RNA-binding domain. |
| DECR1 | Q16698 | 2,4-dienoyl-CoA reductase, mitochondrial | Auxiliary enzyme of beta-oxidation. It participates in the metabolism of unsaturated fatty enoyl-CoA esters having double bonds in both even- and odd-numbered positions. Catalyzes the NADP-dependent reduction of 2,4-dienoyl-CoA to yield trans-3-enoyl-CoA. |
| GRB2 | P62993 | Growth factor receptor-bound protein 2 | Adapter protein that provides a critical link between cell surface growth factor receptors and the Ras signaling pathway. Binds the epidermal growth factor receptor and contains one SH2 domain and two SH3 domains. Its two SH3 domains direct complex formation with proline-rich regions of other proteins, & its SH2 domain binds tyrosine phosphorylated sequences. |
| RBBP7 | Q16576 | RB Binding Protein 7, Chromatin Remodeling Factor | Ubiquitously expressed nuclear protein belonging to a highly conserved subfamily of WD-repeat proteins. Binds directly to retinoblastoma protein, which regulates cell proliferation. Found in many histone deacetylase complexes. Present in protein complexes involved in chromatin assembly. Can interact with BRCA1 tumor-suppressor gene. |
| FARSA | Q9Y285 | Phenylalanine--tRNA ligase alpha subunit | Encodes a product which is similar to the catalytic subunit of prokaryotic and Saccharomyces cerevisiae phenylalanyl-tRNA synthetases (PheRS). This protein is expressed in a tumor-selective and cell cycle stage- and differentiation-dependent manner, the first member of the tRNA synthetase gene family shown to exhibit this type of regulated expression. |
| PDHB | P11177 | Pyruvate Dehydrogenase (Lipoamide) Beta | The pyruvate dehydrogenase complex catalyzes the overall conversion of pyruvate to acetyl-CoA and CO2, links the glycolytic pathway to the tricarboxylic cycle. |
| GMFG | O60234 | Glia maturation factor gamma | No information |
| COTL1 | Q14019 | Coactosin Like F-Actin Binding Protein 1 | Binds to F-actin in a calcium-independent manner. Has no direct effect on actin depolymerization. Acts as a chaperone for ALOX5 (5LO), influencing both its stability & activity in leukotrienes synthesis. Interacts with 5-lipoxygenase, which is the first committed enzyme in leukotriene biosynthesis. |
| EIF4A3 | P38919 | Eukaryotic initiation factor 4A-III | ATP-dependent RNA helicase. Core component of the splicing-dependent multiprotein exon junction complex (EJC) deposited at splice junctions on mRNAs. Shows higher affinity for single-stranded RNA in an ATP-bound core EJC complex than after the ATP is hydrolyzed. Involved in the splicing modulation of BCL2L1/Bcl-X; specifically inhibits formation of proapoptotic isoforms such as Bcl-X(S); the function is different from the established EJC assembly. Involved in craniofacial development. |
| DSP | P15924 | Desmoplakin | Anchors intermediate filaments to desmosomal plaques & forms an obligate component of functional desmosomes. Involved in the organization of the desmosomal cadherin-plakoglobin complexes into discrete plasma membrane domains. |
| RBM14 | Q96PK6 | RNA Binding Motif Protein 14 | Regulates centriole biogenesis by suppressing the formation of aberrant centriolar protein complexes in the cytoplasm, thus preserving mitotic spindle integrity. Prevents the formation of the STIL-CENPJ complex by interfering with the interaction of STIL with CENPJ. |
| LMNB1 | P20700 | Lamin B1 | Component of the nuclear lamina, a fibrous layer on the nucleoplasmic side of the inner nuclear membrane. |

# **Table S2.** Tau interactome enriched in *AKAP9*− group

| **Gene Symbol** | **UniProtKB** | **Gene Name** | **Function (Genecards)** |
| --- | --- | --- | --- |
| IGKC | P01834 | Immunoglobulin kappa constant | Constant region of immunoglobulin heavy chains. Immunoglobulins are membrane-bound or secreted glycoproteins produced by B lymphocytes. In the recognition phase of humoral immunity, the membrane-bound immunoglobulins serve as receptors that, upon binding of a specific antigen, trigger the clonal expansion and differentiation of B lymphocytes into immunoglobulins-secreting plasma cells. |
| PSMD8 | P48556 | 26S proteasome non-ATPase regulatory subunit 8 | Component of the 26S proteasome, a multiprotein complex involved in the ATP-dependent degradation of ubiquitinated proteins. This complex plays a key role in the maintenance of protein homeostasis by removing misfolded or damaged proteins, which could impair cellular functions, and by removing proteins whose functions are no longer required. Therefore, the proteasome participates in numerous cellular processes, including cell cycle progression, apoptosis, or DNA damage repair. |
| HLA-DQA2 | P01906 | HLA class II histocompatibility antigen, DQ alpha 2 chain | Binds peptides derived from antigens that access the endocytic route of antigen presenting cells (APC) and presents them on the cell surface for recognition by the CD4 T-cells. It is located in intracellular vesicles and plays a central role in the peptide loading of MHC class II molecules by helping to release the CLIP molecule from the peptide binding site. Class II molecules are expressed in antigen presenting cells & are used to present antigenic peptides on the cell surface to be recognized by CD4 T-cells. |
| FABP5 | Q01469 | Fatty Acid Binding Protein 5 | High specificity for fatty acids. Highest affinity for C18 chain length. Decreasing the chain length or introducing double bonds reduces the affinity. |
| IGHA1 | P01876 | Immunoglobulin heavy constant alpha 1 | Constant region of immunoglobulin heavy chains. Immunoglobulins, also known as antibodies, are membrane-bound or secreted glycoproteins produced by B lymphocytes. In the recognition phase of humoral immunity, the membrane-bound immunoglobulins serve as receptors which, upon binding of a specific antigen, trigger the clonal expansion & differentiation of B lymphocytes into immunoglobulins-secreting plasma cells. |
| STMN1 | P16949 | Stathmin | Involved in the regulation of the microtubule (MT) filament system by destabilizing microtubules. Prevents assembly & promotes disassembly of microtubules. |
| RPLP1 | P05386 | Ribosomal Protein Lateral Stalk Subunit P1 | Encodes a ribosomal phosphoprotein that is a component of the 60S subunit. The protein belongs to the L12P family of ribosomal proteins. Plays an important role in the elongation step of protein synthesis. Unlike most ribosomal proteins, which are basic, is acidic. Its C-terminal end is nearly identical to the C-terminal ends of the ribosomal phosphoproteins P0 & P2. The protein is located in the cytoplasm. |
| GOT2 | P00505 | Glutamic-Oxaloacetic Transaminase 2 | Glutamic-oxaloacetic transaminase is a pyridoxal phosphate-dependent enzyme that exists in cytoplasmic & inner-membrane mitochondrial forms, GOT1 & GOT2, respectively. GOT plays a role in amino acid metabolism & the urea and tricarboxylic acid cycles. Catalyzes the irreversible transamination of the L-tryptophan metabolite L-kynurenine to form kynurenic acid. Plays a key role in amino acid metabolism. Important for metabolite exchange between mitochondria & cytosol. Facilitates cellular uptake of long-chain free fatty acids. |
| RPL18 | Q07020 | 60S ribosomal protein L18 | This gene encodes a member of the L18E family of ribosomal proteins, component of the 60S subunit. |
| GLUD1 | P00367 | Glutamate dehydrogenase 1, mitochondrial | Mitochondrial glutamate dehydrogenase that converts L-glutamate into alpha-ketoglutarate. Plays a key role in glutamine anaplerosis by producing alpha-ketoglutarate, an important intermediate in the tricarboxylic acid cycle. |
| RPS6 | P62753 | 40S ribosomal protein S6 | Encodes a cytoplasmic ribosomal protein that is a component of the 40S subunit. The protein belongs to the S6E family of ribosomal proteins. It is the major substrate of protein kinases in the ribosome, with subsets of five C-terminal serine residues phosphorylated by different protein kinases. Phosphorylation is induced by a wide range of stimuli, including growth factors, tumor-promoting agents, and mitogens. Dephosphorylation occurs at growth arrest. |
| GBE1 | Q04446 | 1,4-alpha-glucan-branching enzyme | The protein is a glycogen branching enzyme that catalyzes the transfer of alpha-1,4-linked glucosyl units from the outer end of a glycogen chain to an alpha-1,6 position on the same or a neighboring glycogen chain. Branching of the chains is essential to increase the solubility of the glycogen molecule and, consequently, in reducing the osmotic pressure within cells. Highest level of this enzyme are found in liver & muscle. |
| FH | P07954 | Fumarate hydratase, mitochondrial | This protein is an enzymatic component of the tricarboxylic acid cycle and catalyzes the formation of L-malate from fumarate. Exists in both a cytosolic form and an N-terminal extended form, differing only in the translation start site used. The N-terminal extended form is targeted to the mitochondria, where the removal of the extension generates the same form as in the cytoplasm. Acts as a tumor suppressor. |
| VIM | P08670 | Vimentin | Vimentins are class-III intermediate filaments found in various non-epithelial cells. Vimentin is attached to the nucleus, endoplasmic reticulum, and mitochondria, either laterally or terminally and is responsible for maintaining cell shape, integrity of the cytoplasm, and stabilizing cytoskeletal interactions. It functions as an organizer of a number of critical proteins involved in attachment, migration, and cell signaling. Involved with LARP6 in the stabilization of type I collagen mRNAs for CO1A1 and CO1A2. It is involved in the immune response, and controls the transport of low-density lipoprotein-derived cholesterol from a lysosome to the site of esterification. |
| UBE2L3 | P68036 | Ubiquitin Conjugating Enzyme E2 L3 | Ubiquitin-conjugating enzyme E2 that specifically acts with HECT-type and RBR family E3 ubiquitin-protein ligases. it has activity with the RBR family E3 enzymes, such as PARK2 and ARIH1, that function like function like RING-HECT hybrids. Accepts ubiquitin from the E1 complex and catalyzes its covalent attachment to other proteins. Involved in the selective degradation of short-lived and abnormal proteins. Down-regulated during the S-phase it is involved in progression through the cell cycle. |
| ABRACL | Q9P1F3 | ABRA C-Terminal Like | No information |
| RPL36 | Q9Y3U8 | Ribosomal Protein L36 | Encodes a ribosomal protein that is a component of the 60S subunit. The protein belongs to the L36E family of ribosomal proteins. It is located in the cytoplasm. |
| B2M | P61769 | Beta-2-Microglobulin | Component of the class I major histocompatibility complex (MHC) heavy chain on the surface of nearly all nucleated cells. Involved in the presentation of peptide antigens to the immune system. It has a predominantly beta-pleated sheet structure that can form amyloid fibrils in some pathological conditions. The encoded antimicrobial protein displays antibacterial activity in amniotic fluid. |
| MT-CO2 | P00403 | Mitochondrially Encoded Cytochrome C Oxidase II | Cytochrome c oxidase is the component of the respiratory chain that catalyzes the reduction of oxygen to water. Subunits 1-3 form the functional core of the enzyme complex. Subunit 2 transfers the electrons from cytochrome c via its binuclear copper A center to the bimetallic center of the catalytic subunit 1. |
| HSD17B10 | Q99714 | Hydroxysteroid 17-Beta Dehydrogenase 10 | Encodes 3-hydroxyacyl-CoA dehydrogenase type II, a member of the short-chain dehydrogenase/reductase superfamily. The gene produces a mitochondrial protein that catalyzes the oxidation of a wide variety of fatty acids & steroids. Is a subunit of mitochondrial ribonuclease P, which cleaves tRNA molecules in their 5-ends. The protein has been implicated in the development of Alzheimer disease, and mutations in the gene are the cause of 17beta-hydroxysteroid dehydrogenase type 10 (HSD10) deficiency. Catalyzes the beta-oxidation at position 17 of androgens and estrogens and has 3-alpha-hydroxysteroid dehydrogenase activity with androsterone. Catalyzes the third step in the beta-oxidation of fatty acids. Carries out oxidative conversions of 7-alpha-OH and 7-beta-OH bile acids. Exhibits 20-beta-OH and 21-OH dehydrogenase activities with C21 steroids. By interacting with intracellular amyloid-beta, it may contribute to the neuronal dysfunction associated with Alzheimer disease (AD). |
| GFPT1 | Q06210 | Glutamine--Fructose-6-Phosphate Transaminase 1 | This gene encodes the first and rate-limiting enzyme of the hexosamine pathway and controls the flux of glucose into the hexosamine pathway. The product of this gene catalyzes the formation of glucosamine 6-phosphate. Regulates the circadian expression of clock genes ARNTL/BMAL1 and CRY1. |
| ANXA4 | P09525 | Annexin A4 | Calcium/phospholipid-binding protein that promotes membrane fusion & is involved in exocytosis. |
| PGD | P52209 | Phosphogluconate Dehydrogenase | Catalyzes the oxidative decarboxylation of 6-phosphogluconate to ribulose 5-phosphate and CO(2), with concomitant reduction of NADP to NADPH. |
| ANXA7 | P20073 | Annexin A7 | Calcium/phospholipid-binding protein which promotes membrane fusion and is involved in exocytosis. Structural analysis suggests that Annexin VII is a membrane binding protein with diverse properties, including voltage-sensitive calcium channel activity, ion selectivity and membrane fusion. |
| RAB8B | Q92930 | RAB8B, Member RAS Oncogene Family | RAB proteins, like RAB8B, are low molecular mass monomeric GTPases that localize on the cytoplasmic surfaces of distinct membrane-bound organelles. RAB proteins function in intracellular vesicle transport by aiding in the docking and/or fusion of vesicles with their target membranes. |
| RAB1B | Q9H0U4 | RAB1B, Member RAS Oncogene Family | RAB1B regulates vesicular transport between the endoplasmic reticulum and successive Golgi compartments. Plays a role in the initial events of the autophagic vacuole development which take place at specialized regions of the endoplasmic reticulum. |
| WDR1 | O75083 | WD Repeat Domain 1 | Induces disassembly of actin filaments in conjunction with ADF/cofilin family proteins. Involved in cytokinesis. Involved in chemotactic cell migration by restricting lamellipodial membrane protrusions. Involved in myocardium sarcomere organization. Involved in megakaryocyte maturation & platelet shedding. Required for the establishment of planar cell polarity (PCP) during follicular epithelium development & for cell shape changes during PCP. Involved in assembly & maintenance of epithelial apical cell junctions & plays a role in the organization of the perijunctional actomyosin belt. |
| SEPT7 | Q16181 | Septin 7 | Filament-forming cytoskeletal GTPase. Required for normal organization of the actin cytoskeleton. Required for normal progress through mitosis. Involved in cytokinesis. Required for normal association of CENPE with the kinetochore. Plays a role in ciliogenesis and collective cell movements. This protein functions in gliomagenesis and in the suppression of glioma cell growth. |
| RAB8A | P61006 | RAB8A, Member RAS Oncogene Family | Together with RAB11A, RAB3IP, the exocyst complex, PARD3, PRKCI, ANXA2, CDC42 & DNMBP promotes transcytosis of PODXL to the apical membrane initiation sites (AMIS), apical surface formation & lumenogenesis. With MYO5B and RAB11A participates in epithelial cell polarization. Plays an important role in ciliogenesis. |
| CCT4 | P50991 | Chaperonin Containing TCP1 Subunit 4 | Molecular chaperone; assists the folding of proteins upon ATP hydrolysis. Known to play a role, in vitro, in the folding of actin and tubulin. |
| RAB1A | P62820 | RAB1A, Member RAS Oncogene Family | RAB1A regulates vesicular protein transport from the endoplasmic reticulum (ER) to the Golgi compartment and on to the cell surface.Plays a role in IL-8 and growth hormone secretion. Regulates the level of CASR present at the cell membrane. Plays a role in cell adhesion and cell migration, via its role in protein trafficking. Plays a role in autophagosome assembly and cellular defense reactions against pathogenic bacteria. Plays a role in microtubule-dependent protein transport by early endosomes and in anterograde melanosome transport. |
| XRCC5 | P13010 | X-Ray Repair Cross Complementing 5 | Single-stranded DNA-dependent ATP-dependent helicase. Has a role in chromosome translocation. The DNA helicase II complex binds preferentially to fork-like ends of double-stranded DNA in a cell cycle-dependent manner. It works in the 3-5 direction. Involved in DNA non-homologous end joining (NHEJ) required for double-strand break repair and V(D)J recombination. The XRCC5/6 dimer acts as regulatory subunit of the DNA-dependent protein kinase complex DNA-PK by increasing the affinity of the catalytic subunit PRKDC to DNA by 100-fold. The XRCC5/6 dimer together with APEX1 acts as a negative regulator of transcription. |
| SEPT2 | Q15019 | Septin 2 | Filament-forming cytoskeletal GTPase. Required for normal organization of the actin cytoskeleton. Plays a role in the biogenesis of polarized columnar-shaped epithelium by maintaining polyglutamylated microtubules, thus facilitating efficient vesicle transport, and by impeding MAP4 binding to tubulin. Required for progression through mitosis. Forms a scaffold at the midplane of the mitotic splindle required to maintain CENPE localization at kinetochores and consequently chromosome congression. Plays a role in ciliogenesis and collective cell movements. In cilia, required for the integrity of the diffusion barrier at the base of the primary cilium that prevents diffusion of transmembrane proteins between the cilia and plasma membranes. |
| CCT7 | Q99832 | Chaperonin Containing TCP1 Subunit 7 | This gene encodes a molecular chaperone that is a member of the chaperonin containing TCP1 complex (CCT), also known as the TCP1 ring complex (TRiC). This complex consists of two identical stacked rings, each containing 8 different proteins. Unfolded polypeptides enter the central cavity of the complex and are folded in an ATP-dependent manner. The complex folds various proteins, including actin & tubulin. |
| ECH1 | Q13011 | Enoyl-CoA Hydratase 1 | This gene encodes a member of the hydratase/isomerase superfamily. Localizes to the peroxisome. The rat ortholog, which localizes to the matrix of both the peroxisome and mitochondria, can isomerize 3-trans,5-cis-dienoyl-CoA to 2-trans,4-trans-dienoyl-CoA, indicating that it is a delta3,5-delta2,4-dienoyl-CoA isomerase. This enzyme functions in the auxiliary step of the fatty acid beta-oxidation pathway. |
| PSMA4 | P25789 | Proteasome Subunit Alpha 4 | The proteasome is a multicatalytic proteinase complex characterized by its ability to cleave peptides with Arg, Phe, Tyr, Leu, or Glu adjacent to the leaving group at neutral or slightly basic pH. The proteasome has an ATP-dependent proteolytic activity. |
| GSTO1 | P78417 | Glutathione S-Transferase Omega 1 | This protein is an omega class glutathione S-transferase (GST) with glutathione-dependent thiol transferase & dehydroascorbate reductase activities. Involved in the metabolism of xenobiotics & carcinogens. The encoded protein is found in the cytoplasm. Has S-(phenacyl)glutathione reductase activity. Participates in the biotransformation of inorganic arsenic & reduces monomethylarsonic acid (MMA) & dimethylarsonic acid. |
| PSMA1 | P25786 | Proteasome Subunit Alpha 1 | This gene encodes a member of the peptidase T1A family, is a 20S core alpha subunit. |
| RAB14 | P61106 | RAB14, Member RAS Oncogene Family | Involved in membrane trafficking between the Golgi complex and endosomes during early embryonic development. Regulates the Golgi to endosome transport of FGFR-containing vesicles during early development, a key process for developing basement membrane & epiblast and primitive endoderm lineages during early postimplantation development. Regulates, together with guanine nucleotide exchange factor DENND6A, the specific endocytic transport of ADAM10, N-cadherin/CDH2 shedding & cell-cell adhesion. |
| ACAT2 | Q9BWD1 | Acetyl-CoA Acetyltransferase 2 | This is an enzyme involved in lipid metabolism, encodes cytosolic acetoacetyl-CoA thiolase. |
| PSMA2 | P25787 | Proteasome Subunit Alpha 2 | The proteasome is a multicatalytic proteinase complex that is characterized by its ability to cleave peptides with Arg, Phe, Tyr, Leu, or Glu adjacent to the leaving group at neutral or slightly basic pH. The proteasome has ATP-dependent proteolytic activity. |
| APRT | P07741 | Adenine Phosphoribosyltransferase | APRT belongs to the purine/pyrimidine phosphoribosyltransferase family. A conserved feature of this gene is the distribution of CpG dinucleotides. This enzyme catalyzes the formation of AMP and inorganic pyrophosphate from adenine and 5-phosphoribosyl-1-pyrophosphate (PRPP). Produces adenine as a by-product of the polyamine biosynthesis pathway. A homozygous deficiency in this enzyme causes 2,8-dihydroxyadenine urolithiasis. |
| ANP32A | P39687 | Acidic Nuclear Phosphoprotein 32 Family Member A | Implicated in a number of cellular processes, including proliferation, differentiation, caspase-dependent and caspase-independent apoptosis, suppression of transformation (tumor suppressor), inhibition of protein phosphatase 2A, regulation of mRNA trafficking and stability in association with ELAVL1, and inhibition of acetyltransferases as part of the INHAT (inhibitor of histone acetyltransferases) complex. Plays a role in E4F1-mediated transcriptional repression. |
| UBA1 | P22314 | Ubiquitin Like Modifier Activating Enzyme 1 | Catalyzes the first step in ubiquitin conjugation to mark cellular proteins for degradation through the ubiquitin-proteasome system. Activates ubiquitin by first adenylating its C-terminal glycine residue with ATP, and thereafter linking this residue to the side chain of a cysteine residue in E1, yielding a ubiquitin-E1 thioester and free AMP. Essential for the formation of radiation-induced foci, timely DNA repair and for response to replication stress. Promotes the recruitment of TP53BP1 and BRCA1 at DNA damage sites. |
| SHMT2 | P34897 | Serine Hydroxymethyltransferase 2 | Contributes to the de novo mitochondrial thymidylate biosynthesis pathway via its role in glycine and tetrahydrofolate metabolism. Thymidylate biosynthesis is required to prevent uracil accumulation in mtDNA. Interconversion of serine and glycine. Associates with mitochondrial DNA. Plays a role in the deubiquitination of target proteins as component of the BRISC complex. Required for IFNAR1 deubiquitination by the BRISC complex. |
| PNP | P00491 | Purine Nucleoside Phosphorylase | This is an enzyme which reversibly catalyzes the phosphorolysis of purine nucleosides. The purine nucleoside phosphorylases catalyze the phosphorolytic breakdown of the N-glycosidic bond in the beta-(deoxy)ribonucleoside molecules, with the formation of the corresponding free purine bases and pentose-1-phosphate. |
| STIP1 | P31948 | Stress Induced Phosphoprotein 1 | An adaptor protein that coordinates the functions of HSP70 & HSP90 in protein folding. May assist in transfer of proteins from HSP70 to HSP90 by binding both HSP90 & substrate-bound HSP70. Stimulates the ATPase activity of HSP70 & inhibits the ATPase activity of HSP90, suggesting that it regulates both the conformations & ATPase cycles of these chaperones. Co-chaperone for HSP90AA1. |
| RANBP1 | P43487 | RAN Binding Protein 1 | Inhibits GTP exchange on Ran. Forms a Ran-GTP-RANBP1 trimeric complex. Increase GTP hydrolysis induced by the Ran GTPase activating protein RANGAP1. |
| SEPT8 | Q92599 | Septin 8 | Member of the septin family of nucleotide binding proteins, originally described in yeast as cell division cycle regulatory proteins. |
| DSC1 | Q08554 | Desmocollin 1 | Component of intercellular desmosome junctions. Involved in the interaction of plaque proteins & intermediate filaments mediating cell-cell adhesion. |
| PSMB9 | P28065 | Proteasome Subunit Beta 9 | Proteosome subunit involved in antigen processing to generate class I binding peptides. |

# **Table S3.** Ontologies of Tau interactome common to *AKAP9*+ and *AKAP9*− groups

| Category | Term | Count | % | p-Value | Benjamini | Proteins |
| --- | --- | --- | --- | --- | --- | --- |
| UP_KEYWORDS | Acetylation AKAP9- | 39 | 76.50% | 3.00E-20 | 4.10E-18 | GBE1 ABRACL RAB14 RAB1A RAB1B RANBP1 WDR1 XRCC5 ACAT2 APRT ANXA4 ANXA7 CCT4 CCT7 ECH1 FABP5 FH GLUD1 GOT2 GSTO1 HSD17B10 PSMA1 PSMA2 PSMA4 PSMB9 PNP RPL36 RPS6 RPLP1 SEPT2 SEPT7 SEPT8 SHMT2 STIP1 UBE2L3 UBA1 VIM |
|  | Acetylation AKAP9+ | 37 | 72.50% | 5.90E-18 | 8.40E-16 | DECR1 DDX3X EWSR1 FKBP1A RAB11B RAB5B RBBP4 RBBP7 RBM14-RBM4 RBMX RUVBL1 RUVBL2 S100A7 TAF15 C14orf166 COTL1 C1QBP CSTA ERH EIF4A3 GMFG GRB2 HSPB1 HNRNPM LMNB1 LPXN LSP1 FARSA PCMT1 PDHB RPL11 RPL35A SRSF1 SYNGR2 TPM4 USMG5 VDAC2 |
| GOTERM_CC_DIRECT | extracellular exosome AKAP9- | 39 | 76.50% | 5.40E-22 | 8.00E-20 | GBE1 RAB14 RAB1A RAB1B RAB8A RAB8B WDR1 ACAT2 APRT ANXA4 ANXA7 B2M CCT4 CCT7 COX2 DSC1 ECH1 FABP5 FH GOT2 GFPT1 GSTO1 IGHA1 IGKC PGD PSMD8 PSMA1 PSMA2 PSMA4 PSMB9 PNP RPLP1 SEPT2 SEPT7 SHMT2 STMN1 UBE2L3 UBA1 VIM |
|  | extracellular exosome AKAP9+ | 30 | 58.80% | 6.00E-12 | 8.70E-10 | DECR1 CD74 DDX3X FKBP1A RAB11B RAB5B RBMX RUVBL1 RUVBL2 S100A7 ARG1 COTL1 CSTA DSP GRB2 HSPB1 HNRNPM KRT33B LSP1 PCMT1 PDHB RHOG RPL11 RPL35A SRSF1 SSR4 SYNGR2 TPM4 USMG5 VDAC2 |
| GOTERM_MF_DIRECT | poly(A) RNA binding AKAP9- | 11 | 21.60% | 1.10E-03 | 4.10E-02 | XRCC5 ANP32A ANXA7 CCT4 GOT2 HSD17B10 RPL36 RPS6 STIP1 UBE2L3 UBA1 |
|  | poly(A) RNA binding AKAP9+ | 18 | 35.30% | 5.60E-09 | 8.20E-07 | DDX3X EWSR1 RBM14-RBM4 RBMX TAF15 C14orf166 DSP ERH EIF4A3 GRB2 small HSPB1 HNRNPM FARSA PRMT1 RPL11 RPL35A SRSF1 SNRPD1 |
| UP_KEYWORDS | Cytoplasm AKAP9- | 31 | 60.80% | 2.00E-06 | 1.00E-04 | RANBP1 WDR1 ACAT2 ANP32A APRT ANXA4 B2M CCT4 CCT7 FABP5 FH GLUD1 GSTO1 HSD17B10 PSMA1 PSMA2 PSMA4 PSMB9 PNP RPL18 RPL36 RPS6 RPLP1 SEPT2 SEPT7 SEPT8 SHMT2 STMN1 UBE2L3 UBA1 VIM |
|  | Cytoplasm AKAP9+ | 24 | 47.10% | 3.70E-04 | 6.60E-03 | DDX3X EWSR1 FKBP1A RBM14-RBM4 RUVBL1 RUVBL2 S100A7 TAF15 ARG1 C14orf166 COTL1 C1QBP CSTA DSP EIF4A3 GRB2 HSPB1 LPXN FARSA PRMT1 PCMT1 SRSF1 SNRPD1 TPM4 |
| UP_KEYWORDS | Phosphoprotein AKAP9- | 37 | 72.50% | 4.70E-06 | 1.60E-04 | GBE1 RAB1A RAB1B RAB8A RAB8B RANBP1 WDR1 XRCC5 ANP32A APRT ANXA4 CCT4 DSC1 ECH1 FABP5 FH GLUD1 GOT2 GFPT1 GSTO1 PGD PSMD8 PSMA1 PSMA2 PSMA4 PNP RPL18 RPS6 RPLP1 SEPT2 SEPT7 SEPT8 SHMT2 STMN1 STIP1 UBA1 VIM |
|  | Phosphoprotein AKAP9+ | 32 | 62.70% | 1.40E-03 | 1.80E-02 | DECR1 CD74 DDX3X Hsp40 DNAJB11 EWSR1 KIAA0391 RBBP4 RBBP7 RBM14-RBM4 RBMX RUVBL2 TAF15 ARG1 C1QBP DHPS DSP ERH EIF4A3 GRB2 HSPB1 HNRNPM LMNB1 LPXN LSP1 FARSA PRMT1 PDHB RHOG RPL11 SRSF1 SYNGR2 TPM4 |
| GOTERM_CC_DIRECT | cytoplasm AKAP9- | 31 | 60.80% | 2.00E-06 | 1.00E-04 | RANBP1 WDR1 ACAT2 ANP32A APRT ANXA4 B2M CCT4 CCT7 FABP5 FH GLUD1 GSTO1 HSD17B10 PSMA1 PSMA2 PSMA4 PSMB9 PNP RPL18 RPL36 RPS6 RPLP1 SEPT2 SEPT7 SEPT8 SHMT2 STMN1 UBE2L3 UBA1 VIM |
|  | cytoplasm AKAP9+ | 25 | 49.00% | 2.90E-03 | 4.10E-02 | DECR1 DDX3X EWSR1 FKBP1A RBM14-RBM4 RUVBL1 RUVBL2 S100A7 TAF15 ARG1 C14orf166 COTL1 C1QBP CSTA DHPS DSP EIF4A3 GRB2 HSPB1 LPXN FARSA PRMT1 PCMT1 RPL11 SRSF1 |

# **Table S4.** Ontologies of Tau interactome enriched in *AKAP9*+ group

| Category | Term | Count | % | p-Value | Benjamini | Proteins |
| --- | --- | --- | --- | --- | --- | --- |
| UP_KEYWORDS | RNA-binding | 12 | 23.50% | 4.40E-07 | 3.20E-05 | DDX3X EWSR1 RBM14-RBM4 RBMX TAF15 C14orf166 EIF4A3 HNRNPM RPL11 RPL35A SRSF1 SNRPGP15 |
| GOTERM_MF_DIRECT | RNA binding | 11 | 21.60% | 2.50E-06 | 1.90E-04 | DDX3X EWSR1 RBBP7 RBM14-RBM4 RBMX C14orf166 HNRNPM RPL11 SRSF1 SNRPD1 SNRPGP15 |
| GOTERM_CC_DIRECT | membrane | 19 | 37.30% | 1.10E-05 | 7.90E-04 | CD74 DNAJB1\1 FKBP1A RAB5B RBMX RUVBL1 RUVBL2 C1QBP ERH EIF4A3 HNRNPM LMNB1 LPXN LSP1 HLA-DPB1 FARSA RPL11 RPL35A TPM4 |
| UP_KEYWORDS | Isopeptide bond | 13 | 25.50% | 1.30E-05 | 6.00E-04 | DDX3X RBBP4 RBBP7 RBM14-RBM4 RBMX RUVBL1 ERH EIF4A3 HNRNPM LMNB1 SRSF1 SSR4 VDAC2 |
| UP_KEYWORDS | Spliceosome | 6 | 11.80% | 1.40E-05 | 5.00E-04 | RBMX EIF4A3 HNRNPM SRSF1 SNRPD1 SNRPGP15 |
| UP_KEYWORDS | Methylation | 12 | 23.50% | 2.20E-05 | 6.40E-04 | DDX3X EWSR1 RAB11B RBMX TAF15 DSP HSPB1 HNRNPM LMNB1 RHOG SRSF1 SNRPD1 |
| UP_KEYWORDS | mRNA splicing | 7 | 13.70% | 3.80E-05 | 9.20E-04 | RBMX C1QBP EIF4A3 HNRNPM SRSF1 SNRPD1 SNRPGP15 |
| GOTERM_MF_DIRECT | protein binding | 39 | 76.50% | 9.40E-05 | 4.60E-03 | CD74 DDX3X DNAJB11 EWSR1 FKBP1A RAB11B RAB5B RBBP4 RBBP7 RBMX RUVBL1 RUVBL2 S100A7 TAF15 C14orf166 COTL1 C1QBP DHPS DSP ERH EIF4A3 GRB2 HSPB1 HNRNPM KRT33B LMNB1 LPXN FARSA PRMT1 PCMT1 PDHB RHOG RPL11 RPL35A SRSF1 SNRPD1 SYNGR2 TPM4 VDAC2 |
| GOTERM_CC_DIRECT | catalytic step 2 spliceosome | 5 | 9.80% | 1.20E-04 | 5.70E-03 | RBMX EIF4A3 HNRNPM SRSF1 SNRPD1 |
| UP_KEYWORDS | mRNA processing | 7 | 13.70% | 1.50E-04 | 3.00E-03 | RBMX C1QBP EIF4A3 HNRNPM SRSF1 SNRPD1 SNRPGP15 |
| GOTERM_CC_DIRECT | extracellular matrix | 7 | 13.70% | 1.50E-04 | 5.50E-03 | FKBP1A CSTA DSP HSPB1 HNRNPM RPL11 RPL35A |
| GOTERM_MF_DIRECT | nucleotide binding | 7 | 13.70% | 4.30E-04 | 1.60E-02 | EWSR1 RBM14-RBM4 RBMX TAF15 HNRNPM SRSF1 VDAC2 |
| GOTERM_CC_DIRECT | methylosome | 3 | 5.90% | 4.80E-04 | 1.40E-02 | ERH PRMT1 SNRPD1 |
| GOTERM_CC_DIRECT | intracellular ribonucleoprotein complex | 5 | 9.80% | 5.20E-04 | 1.30E-02 | RBM14-RBM4 RBMX EIF4A3 HNRNPM SNRPD1 |
| UP_KEYWORDS | Ubl conjugation | 13 | 25.50% | 6.30E-04 | 1.00E-02 | DDX3X RBBP4 RBBP7 RBM14-RBM4 RBMX RUVBL1 ERH EIF4A3 HNRNPM LMNB1 SRSF1 SSR4 VDAC2 |
| UP_KEYWORDS | Ribonucleo-protein | 6 | 11.80% | 7.40E-04 | 1.10E-02 | RBMX HNRNPM RPL11 RPL35A SNRPD1 SNRPGP15 |
| GOTERM_CC_DIRECT | nucleoplasm | 18 | 35.30% | 8.00E-04 | 1.70E-02 | DECR1 RBBP4 RBBP7 RBM14-RBM4 RBMX RUVBL1 RUVBL2 TAF15 C14orf166 EIF4A3 GRB2 HNRNPM LMNB1 PRMT1 PDHB RPL11 SRSF1 SNRPD1 |
| GOTERM_MF_DIRECT | identical protein binding | 9 | 17.60% | 1.10E-03 | 3.30E-02 | CD74 EWSR1 RBMX RUVBL2 C14orf166 DHPS GRB2 HSPB1 PRMT1 |
| UP_KEYWORDS | Chaperone | 5 | 9.80% | 1.40E-03 | 1.70E-02 | CD74 DNAJB11 RBBP7 COTL1 HSPB1 |
| GOTERM_CC_DIRECT | nucleus | 26 | 51.00% | 2.00E-03 | 3.60E-02 | DECR1DDX3X DNAJB11 EWSR1 RBBP4 RBBP7 RBM14-RBM4 RBMX RUVBL1 RUVBL2 S100A7 TAF15 ARG1 C14orf166 COTL1 C1QBP CSTA DSP GRB2 HSPB1 LPXN PRMT1 PDHB SRSF1 SNRPD1 VDAC2 |
| GOTERM_CC_DIRECT | nuclear matrix | 4 | 7.80% | 2.40E-03 | 3.80E-02 | RUVBL1 RUVBL2 HNRNPM LMNB1 |
| UP_KEYWORDS | Nucleus | 23 | 45.10% | 3.30E-03 | 3.60E-02 | DDX3X EWSR1 RBBP4 RBBP7 RBM14-RBM4 RBMX RUVBL1 RUVBL2 TAF15 C14orf166 COTL1 C1QBP EIF4A3 GRB2 HSPB1 HNRNPM LMNB1 LPXN PRMT1 RPL11 SRSF1 SNRPD1 SNRPGP15 |

# **Table S5.** Ontologies of Tau interactome enriched in *AKAP9*− group

| Category | Term | Count | % | p-Value | Benjamini | Proteins |
| --- | --- | --- | --- | --- | --- | --- |
| GOTERM_CC_DIRECT | cytosol | 29 | 56.90% | 1.20E-09 | 8.90E-08 | GBE1 RAB14 RAB1A RAB8A WDR1 XRCC5 APRT ANXA7 CCT4 CCT7 FABP5 FH GFPT1 GSTO1 PGD PSMD8 PSMA1 PSMA2 PSMA4 PSMB9 PNP RPL18 RPL36 RPS6 RPLP1 SEPT7 STMN1 UBA1 VIM |
| GOTERM_BP_DIRECT | antigen processing and presentation of exogenous peptide antigen via MHC class I, TAP-dependent | 6 | 11.80% | 9.60E-07 | 3.40E-04 | B2M PSMD8 PSMA1 PSMA2 PSMA4 PSMB9 |
| UP_KEYWORDS | GTP-binding | 9 | 17.60% | 1.60E-06 | 1.10E-04 | RAB14 RAB1A RAB1B RAB8A RAB8B GLUD1 SEPT2 SEPT7 SEPT8 |
| UP_KEYWORDS | Proteasome | 5 | 9.80% | 8.30E-06 | 2.30E-04 | PSMD8 PSMA1 PSMA2 PSMA4 PSMB9 |
| GOTERM_MF_DIRECT | GTP binding | 9 | 17.60% | 1.10E-05 | 1.30E-03 | RAB14 RAB1A RAB1B RAB8A RAB8B GLUD1 SEPT2 SEPT7 SEPT8 |
| GOTERM_BP_DIRECT | regulation of mRNA stability | 6 | 11.80% | 1.10E-05 | 2.00E-03 | ANP32A PSMD8 PSMA1 PSMA2 PSMA4 PSMB9 |
| GOTERM_BP_DIRECT | regulation of cellular amino acid metabolic process | 5 | 9.80% | 1.30E-05 | 1.60E-03 | PSMD8 PSMA1 PSMA2 PSMA4 PSMB9 |
| UP_KEYWORDS | Threonine protease | 4 | 7.80% | 1.50E-05 | 3.40E-04 | PSMA1 PSMA2 PSMA4 PSMB9 |
| GOTERM_CC_DIRECT | proteasome complex | 5 | 9.80% | 2.00E-05 | 7.40E-04 | PSMD8 PSMA1 PSMA2 PSMA4 PSMB9 |
| GOTERM_CC_DIRECT | proteasome core complex | 4 | 7.80% | 2.30E-05 | 6.90E-04 | PSMA1 PSMA2 PSMA4 PSMB9 |
| GOTERM_MF_DIRECT | threonine-type endopeptidase activity | 4 | 7.80% | 2.80E-05 | 1.60E-03 | PSMA1 PSMA2 PSMA4 PSMB9 |
| GOTERM_BP_DIRECT | NIK/NF-kappaB signaling | 5 | 9.80% | 3.70E-05 | 3.30E-03 | PSMD8 PSMA1 PSMA2 PSMA4 PSMB9 |
| GOTERM_BP_DIRECT | negative regulation of ubiquitin-protein ligase activity involved in mitotic cell cycle | 5 | 9.80% | 5.00E-05 | 3.50E-03 | PSMD8 PSMA1 PSMA2 PSMA4 PSMB9 |
| GOTERM_BP_DIRECT | T cell receptor signaling pathway | 6 | 11.80% | 6.30E-05 | 3.70E-03 | HLA-DQA2 PSMD8 PSMA1 PSMA2 PSMA4 PSMB9 |
| GOTERM_BP_DIRECT | positive regulation of ubiquitin-protein ligase activity involved in regulation of mitotic cell cycle transition | 5 | 9.80% | 6.50E-05 | 3.30E-03 | PSMD8 PSMA1 PSMA2 PSMA4 PSMB9 |
| GOTERM_BP_DIRECT | anaphase-promoting complex-dependent catabolic process | 5 | 9.80% | 7.50E-05 | 3.40E-03 | PSMD8 PSMA1 PSMA2 PSMA4 PSMB9 |
| KEGG_PATHWAY | Proteasome | 5 | 9.80% | 9.00E-05 | 8.20E-03 | PSMD8 PSMA1 PSMA2 PSMA4 PSMB9 |
| GOTERM_BP_DIRECT | Wnt signaling pathway, planar cell polarity pathway | 5 | 9.80% | 1.40E-04 | 5.40E-03 | PSMD8 PSMA1 PSMA2 PSMA4 PSMB9 |
| GOTERM_BP_DIRECT | Fc-epsilon receptor signaling pathway | 6 | 11.80% | 1.50E-04 | 5.30E-03 | IGKC PSMD8 PSMA1 PSMA2 PSMA4 PSMB9 |
| GOTERM_BP_DIRECT | protein polyubiquitination | 6 | 11.80% | 1.70E-04 | 5.70E-03 | PSMD8 PSMA1 PSMA2 PSMA4 PSMB9 UBE2L3 |
| GOTERM_CC_DIRECT | proteasome core complex, alpha-subunit complex | 3 | 5.90% | 2.00E-04 | 4.80E-03 | PSMA1 PSMA2 PSMA4 |
| GOTERM_BP_DIRECT | stimulatory C-type lectin receptor signaling pathway | 5 | 9.80% | 2.30E-04 | 6.80E-03 | PSMD8 PSMA1 PSMA2 PSMA4 PSMB9 |
| UP_KEYWORDS | Nucleotide-binding | 14 | 27.50% | 2.60E-04 | 5.00E-03 | RAB14 RAB1A RAB1B RAB8A RAB8B XRCC5 CCT4 CCT7 GLUD1 SEPT2 SEPT7 SEPT8 UBE2L3 UBA1 |
| GOTERM_BP_DIRECT | proteolysis involved in cellular protein catabolic process | 4 | 7.80% | 3.50E-04 | 9.50E-03 | PSMA1 PSMA2 PSMA4 PSMB9 |
| KEGG_PATHWAY | Carbon metabolism | 6 | 11.80% | 3.50E-04 | 1.60E-02 | ACAT2 FH GLUD1 GOT2 PGD SHMT2 |
| GOTERM_BP_DIRECT | tumor necrosis factor-mediated signaling pathway | 5 | 9.80% | 3.60E-04 | 9.00E-03 | PSMD8 PSMA1 PSMA2 PSMA4 PSMB9 |
| GOTERM_BP_DIRECT | positive regulation of canonical Wnt signaling pathway | 5 | 9.80% | 3.80E-04 | 9.00E-03 | PSMD8 PSMA1 PSMA2 PSMA4 PSMB9 |
| GOTERM_BP_DIRECT | cilium morphogenesis | 5 | 9.80% | 6.10E-04 | 1.40E-02 | RAB1A RAB8A RAB8B SEPT2 SEPT7 |
| GOTERM_CC_DIRECT | mitochondrion | 12 | 23.50% | 6.70E-04 | 1.40E-02 | RAB1B RAB8B ACAT2 CCT7 COX2 ECH1 FH GLUD1 GOT2 HSD17B10 SHMT2 UBA1 |
| GOTERM_CC_DIRECT | myelin sheath | 5 | 9.80% | 7.40E-04 | 1.30E-02 | WDR1 GOT2 SEPT2 SEPT8 STIP1 |
| UP_KEYWORDS | Prenylation | 5 | 9.80% | 7.40E-04 | 1.30E-02 | RAB14 RAB1A RAB1B RAB8A RAB8B |
| KEGG_PATHWAY | Biosynthesis of antibiotics | 7 | 13.70% | 9.40E-04 | 2.80E-02 | ACAT2 FH GOT2 GFPT1 HSD17B10 PGD SHMT2 |
| GOTERM_BP_DIRECT | negative regulation of canonical Wnt signaling pathway | 5 | 9.80% | 1.20E-03 | 2.50E-02 | PSMD8 PSMA1 PSMA2 PSMA4 PSMB9 |
| GOTERM_CC_DIRECT | nonmotile primary cilium | 3 | 5.90% | 1.70E-03 | 2.80E-02 | RAB8A SEPT2 SEPT7 |
| GOTERM_BP_DIRECT | ubiquitin-dependent protein catabolic process | 5 | 9.80% | 1.80E-03 | 3.50E-02 | RANBP1 PSMA1 PSMA2 PSMA4 UBE2L3 |
| GOTERM_BP_DIRECT | SRP-dependent cotranslational protein targeting to membrane | 4 | 7.80% | 2.40E-03 | 4.50E-02 | RPL18 RPL36 RPS6 RPLP1 |
| GOTERM_BP_DIRECT | proteasome-mediated ubiquitin-dependent protein catabolic process | 5 | 9.80% | 2.70E-03 | 4.70E-02 | PSMD8 PSMA1 PSMA2 PSMA4 PSMB9 |

# **Table S6.** Tau interactome unique to AD+ group

| **Gene Symbol** | **UniProtKB** | **Gene Name** | **Function (GeneCards)** |
| --- | --- | --- | --- |
| SLIRP | Q9GZT3 | SRA Stem-Loop Interacting RNA Binding Protein | Steroid receptor RNA activator (SRA) is a complex RNA molecule containing multiple stable stem-loop structures that function in coactivation of nuclear receptors. SLIRP interacts with stem-loop structure-7 of SRA (STR7) & modulates nuclear receptor transactivation. RNA-binding protein that acts as a nuclear receptor corepressor. Probably binds the SRA RNA, repressing the SRA-mediated nuclear receptor coactivation. Binds the STR7 loop of SRA RNA. Able to repress glucocorticoid, androgen, thyroid & VDR-mediated transactivation. |
| FKBP1A | P62942 | FK506 Binding Protein 1A | A cis-trans prolyl isomerase that binds the immunosuppressants FK506 & rapamycin. Interacts with several intracellular signal transduction proteins including type I TGF-beta receptor & multiple intracellular calcium release channels. Coordinates multi-protein complex formation of the tetrameric skeletal muscle ryanodine receptor. Keeps in an inactive conformation TGFBR1, the TGF-beta type I serine/threonine kinase receptor, preventing TGF-beta receptor activation in absence of ligand. Recruits SMAD7 to ACVR1B, preventing the association of SMAD2 & SMAD3 with the activin receptor complex, thereby blocking the activin signal. PPIases accelerate the folding of proteins. Catalyzes the cis-trans isomerization of proline imidic peptide bonds in oligopeptides. |
| PSMD8 | P48556 | Proteasome 26S Subunit, Non-ATPase 8 | A non-ATPase subunit of the 19S regulator. A regulatory subunit of the 26S proteasome that is involved in the ATP-dependent degradation of ubiquitinated proteins. Necessary for activation of the CDC28 kinase. |
| IGHG1 | P01857 | Immunoglobulin Heavy Constant Gamma 1 (G1m Marker) | No information |
| GOT2 | P00505 | Glutamic-Oxaloacetic Transaminase 2 | Glutamic-oxaloacetic transaminase is a pyridoxal phosphate-dependent enzyme that exists in cytoplasmic & inner-membrane mitochondrial forms, GOT1 & GOT2, respectively. GOT plays a role in amino acid metabolism & the urea & tricarboxylic acid cycles. Catalyzes the irreversible transamination of the L-tryptophan metabolite L-kynurenine to form kynurenic acid. Plays a key role in amino acid metabolism. Important for metabolite exchange between mitochondria & cytosol. Facilitates cellular uptake of long-chain free fatty acids. |
| GLUD1 | P00367 | Glutamate Dehydrogenase 1 | Mitochondrial glutamate dehydrogenase that converts L-glutamate into alpha-ketoglutarate. Plays a key role in glutamine anaplerosis by producing alpha-ketoglutarate, an important intermediate in the tricarboxylic acid cycle. |
| RPL18 | Q07020 | Ribosomal Protein L18 | Member of the L18E family of ribosomal proteins, component of the 60S subunit. |
| GBE1 | Q04446 | 1,4-Alpha-Glucan Branching Enzyme 1 | Glycogen branching enzyme that catalyzes the transfer of alpha-1,4-linked glucosyl units from the outer end of a glycogen chain to an alpha-1,6 position on the same or a neighboring glycogen chain. Branching of the chains is essential to increase the solubility of the glycogen molecule & in reducing the osmotic pressure within cells. Highest levels of this enzyme are found in liver and muscle. |
| FH | P07954 | Fumarate Hydratase | Enzymatic component of the tricarboxylic acid (TCA) cycle. Catalyzes the formation of L-malate from fumarate. Exists in both a cytosolic form & N-terminal extended form, differing only in the translation start site used. The N-terminal extended form is targeted to the mitochondrion, where the removal of the extension generates the same form as in the cytoplasm. A tumor suppressor. |
| ERP29 | P30040 | Endoplasmic Reticulum Protein 29 | A reticuloplasmin protein; resides in the lumen of the endoplasmic reticulum (ER). Shows sequence similarity to the protein disulfide isomerase family but lacks the thioredoxin motif, suggesting that it does not function as a disulfide isomerase. Thought to play a role in the processing of secretory proteins within the ER, possibly by participating in the folding of proteins there. |
| UBE2I | P63279 | Ubiquitin Conjugating Enzyme E2 I | A member of the E2 ubiquitin-conjugating enzyme family. Can catalyze the formation of poly-SUMO chains. Necessary for sumoylation of FOXL2 & KAT5. Essential for nuclear architecture & chromosome segregation. Sumoylates p53/TP53 at Lys-386. |
| SEPT11 | Q9NVA2 | Septin 11 | Filament-forming cytoskeletal GTPase. |
| ANXA7 | P20073 | Annexin A7 | Calcium/phospholipid-binding protein that promotes membrane fusion & is involved in exocytosis. Likely is a membrane binding protein with diverse properties, including voltage-sensitive calcium channel activity, ion selectivity & membrane fusion. |
| HSD17B10 | Q99714 | Hydroxysteroid 17-Beta Dehydrogenase 10 | A 3-hydroxyacyl-CoA dehydrogenase type II, member of the short-chain dehydrogenase/reductase superfamily. A mitochondrial protein that catalyzes the oxidation of a variety of fatty acids & steroids. Is a subunit of mitochondrial ribonuclease P, which cleaves tRNA molecules in their 5-ends. By interacting with intracellular amyloid-beta, may contribute to neuronal dysfunction associated with Alzheimer disease (AD). Catalyzes the beta-oxidation at position 17 of androgens & estrogens. Has 3-alpha-hydroxysteroid dehydrogenase activity with androsterone. Catalyzes the third step in the beta-oxidation of fatty acids. Carries out oxidative conversions of 7-alpha-OH and 7-beta-OH bile acids. Exhibits 20-beta-OH & 21-OH dehydrogenase activities with C21 steroids. |
| WDR1 | O75083 | WD Repeat Domain 1 | Induces disassembly of actin filaments in conjunction with ADF/cofilin family proteins. Involved in cytokinesis. Involved in chemotactic cell migration by restricting lamellipodial membrane protrusions. Involved in myocardium sarcomere organization. Involved in megakaryocyte maturation & platelet shedding. Required for the establishment of planar cell polarity (PCP) during follicular epithelium development & for cell shape changes during PCP. Involved in assembly & maintenance of epithelial apical cell junctions & in organization of the perijunctional actomyosin belt. |
| SEPT7 | Q16181 | Septin 7 | Filament-forming cytoskeletal GTPase. Required for normal organization of the actin cytoskeleton. Required for normal progress through mitosis. Involved in cytokinesis. Required for normal association of CENPE with the kinetochore. Plays a role in ciliogenesis & collective cell movements. Functions in gliomagenesis & in suppression of glioma cell growth. |
| SLC25A3 | Q00325 | Solute Carrier Family 25 Member 3 | Catalyzes the transport of phosphate into the mitochondrial matrix, either by proton cotransport or in exchange for hydroxyl ions. |
| RPS6 | P62753 | Ribosomal Protein S6 | Encodes a cytoplasmic ribosomal protein component of the 40S subunit. Belongs to the S6E family of ribosomal proteins. The major substrate of protein kinases in the ribosome, with subsets of five C-terminal serine residues phosphorylated by different protein kinases. Phosphorylation is induced by a wide range of stimuli, including growth factors, tumor-promoting agents, & mitogens. Dephosphorylation occurs at growth arrest. |
| PARP1 | P09874 | Poly(ADP-Ribose) Polymerase 1 | A chromatin-associated enzyme, poly(ADP-ribosyl)transferase, which modifies various nuclear proteins by poly(ADP-ribosyl)ation. The modification is dependent on DNA & is involved in the regulation of various important cellular processes such as differentiation, proliferation, & tumor transformation & molecular events involved in the recovery of cell from DNA damage. Involved in the base excision repair (BER) pathway. Positively regulates the transcription of MTUS1, negatively regulates the transcription of MTUS2/TIP150. With EEF1A1 & TXK, forms a complex that acts as a T-helper 1 cell-specific transcription factor & binds the promoter of IFN-gamma to directly regulate its transcription, involved in Th1 cytokine production. Required for PARP9 & DTX3L recruitment to DNA damage sites. Mediates the poly(ADP-ribosyl)ation of histones in a HPF1-dependent manner. Involved in the synthesis of ATP in the nucleus. |
| PGD | P52209 | Phosphogluconate Dehydrogenase | Catalyzes the oxidative decarboxylation of 6-phosphogluconate to ribulose 5-phosphate and CO(2), with concomitant reduction of NADP to NADPH. |
| ECH1 | Q13011 | Enoyl-CoA Hydratase 1 | Member of the hydratase/isomerase superfamily. Localizes to the peroxisome. The rat ortholog, which localizes to the matrix of both the peroxisome & mitochondria, can isomerize 3-trans,5-cis-dienoyl-CoA to 2-trans,4-trans-dienoyl-CoA, indicating that it is a delta3,5-delta2,4-dienoyl-CoA isomerase. Functions in the auxiliary step of the fatty acid beta-oxidation pathway. |
| CCT4 | P50991 | Chaperonin Containing TCP1 Subunit 4 | Molecular chaperone; assists the folding of proteins upon ATP hydrolysis. Known to play a role, in vitro, in the folding of actin & tubulin. |
| PSMB1 | P20618 | Proteasome Subunit Beta 1 | Member of the proteasome B-type family (T1B family) that is a 20S core beta subunit. The gene is tightly linked to the TATA-binding protein gene. |
| XRCC5 | P13010 | X-Ray Repair Cross Complementing 5 | Single-stranded DNA-dependent ATP-dependent helicase. Has a role in chromosome translocation. Binds preferentially to fork-like ends of double-stranded DNA in a cell cycle-dependent manner. Works in the 3-5 direction. Involved in DNA non-homologous end joining (NHEJ) required for double-strand break repair & V(D)J recombination. The XRCC5/6 dimer acts as regulatory subunit of the DNA-dependent protein kinase complex DNA-PK by increasing the affinity of the catalytic subunit PRKDC to DNA by 100-fold. The XRCC5/6 dimer with APEX1 acts as a negative regulator of transcription. |
| PSMA1 | P25786 | Proteasome Subunit Alpha 1 | A member of the peptidase T1A family, is a 20S core alpha subunit. |
| APRT | P07741 | Adenine Phosphoribosyltransferase | Belongs to the purine/pyrimidine phosphoribosyltransferase family. A conserved feature of this gene is the distribution of CpG dinucleotides. Catalyzes the formation of AMP & inorganic pyrophosphate from adenine and 5-phosphoribosyl-1-pyrophosphate (PRPP). Produces adenine as a by-product of the polyamine biosynthesis pathway. Homozygous deficiency in this enzyme causes 2,8-dihydroxyadenine urolithiasis. |
| ANXA4 | P09525 | Annexin A4 | Calcium/phospholipid-binding protein that promotes membrane fusion & is involved in exocytosis. |
| GSTO1 | P78417 | Glutathione S-Transferase Omega 1 | An omega class glutathione S-transferase (GST) with glutathione-dependent thiol transferase & dehydroascorbate reductase activities. GSTs are involved in the metabolism of xenobiotics & carcinogens. Found in the cytoplasm. Has S-(phenacyl)glutathione reductase activity. Participates in the biotransformation of inorganic arsenic & reduces monomethylarsonic acid & dimethylarsonic acid. |
| CANX | P27824 | Calnexin | A member of the calnexin family of molecular chaperones. Is a calcium-binding, endoplasmic reticulum-associated protein that interacts transiently with newly synthesized N-linked glycoproteins, facilitating protein folding & assembly. |
| SEPT2 | Q15019 | Septin 2 | Filament-forming cytoskeletal GTPase. Required for normal organization of the actin cytoskeleton. Plays a role in the biogenesis of polarized columnar-shaped epithelium by maintaining polyglutamylated microtubules, facilitating efficient vesicle transport, & by impeding MAP4 binding to tubulin. Required for progression through mitosis. Forms a scaffold at the midplane of the mitotic splindle required to maintain CENPE localization at kinetochores & consequently chromosome congression. Plays a role in ciliogenesis & collective cell movements. In cilia, required for the integrity of the diffusion barrier at the base of the primary cilium that prevents diffusion of transmembrane proteins between the cilia and plasma membranes. |
| ACAT2 | Q9BWD1 | Acetyl-CoA Acetyltransferase 2 | An enzyme involved in lipid metabolism, cytosolic acetoacetyl-CoA thiolase. |
| CCT7 | Q99832 | Chaperonin Containing TCP1 Subunit 7 | A molecular chaperone member of the chaperonin containing TCP1 complex (CCT), also known as the TCP1 ring complex (TRiC). This complex consists of 2 identical stacked rings, each containing 8 different proteins. Unfolded polypeptides enter the central cavity of the complex & are folded in an ATP-dependent manner. The complex folds various proteins, including actin & tubulin. |
| SEPT8 | Q92599 | Septin 8 | Member of the septin family of nucleotide binding proteins, originally described in yeast as cell division cycle regulatory proteins. |
| RPL36 | Q9Y3U8 | Ribosomal Protein L36 | Ribosomal protein component of the 60S subunit. Belongs to the L36E family of ribosomal proteins. Located in the cytoplasm. |
| EHD1 | Q9H4M9 | EH Domain Containing 1 | ATP- & membrane-binding protein that controls membrane reorganization/tubulation upon ATP hydrolysis. Acts in early endocytic membrane fusion & membrane trafficking of recycling endosomes. Plays a role in the formation of the ciliary vesicle (CV), an early step in cilium biogenesis. May be required for the fusion of distal appendage vesicles (DAVs) to form the CV by recruiting SNARE complex component SNAP29. Required for recruitment of transition zone proteins CEP290, RPGRIP1L, TMEM67 & B9D2, & of IFT20 following DAV reorganization before Rab8-dependent ciliary membrane extension. Required for the loss of CCP110 from the mother centriole needed for the maturation of the basal body during ciliogenesis. |
| VDAC2 | P45880 | Voltage Dependent Anion Channel 2 | Forms a channel through the mitochondrial outer membrane allowing diffusion of small hydrophilic molecules. The channel adopts an open conformation at low or 0 membrane potential & a closed conformation at potentials > 30-40 mV. The open state has a weak anion selectivity, but the closed state is cation-selective. |
| RPN1 | P04843 | Ribophorin I | Type I integral membrane protein found only in the rough endoplasmic reticulum. Part of an N-oligosaccharyl transferase complex linking high mannose oligosaccharides to asparagine residues in the Asn-X-Ser/Thr consensus motif of nascent polypeptide chains. A regulatory subunit of the 26S proteasome. |
| RAB8A | P61006 | RAB8A, Member RAS Oncogene Family | With RAB11A, RAB3IP, the exocyst complex, PARD3, PRKCI, ANXA2, CDC42 & DNMBP promotes transcytosis of PODXL to the apical membrane initiation sites, apical surface formation & lumenogenesis. With MYO5B & RAB11A, participates in epithelial cell polarization. Plays an important role in ciliogenesis. |
| ESD | P10768 | Esterase D | A serine hydrolase belonging to the esterase D family. Active toward numerous substrates including O-acetylated sialic acids. Involved in the detoxification of formaldehyde. |
| STIP1 | P31948 | Stress Induced Phosphoprotein 1 | An adaptor protein that coordinates the functions of HSP70 & HSP90 in protein folding. May assist transfer of proteins from HSP70 to HSP90 by binding both HSP90 & substrate-bound HSP70. Stimulates the ATPase activity of HSP70. Inhibits the ATPase activity of HSP90, perhaps regulating both the conformations & ATPase cycles of these chaperones. Co-chaperone for HSP90AA1. |
| UBA1 | P22314 | Ubiquitin Like Modifier Activating Enzyme 1 | Catalyzes the first step in ubiquitin conjugation to mark cellular proteins for degradation via the ubiquitin-proteasome system. Activates ubiquitin by first adenylating its C-terminal glycine residue with ATP, & thereafter linking this residue to the side chain of a cysteine residue in E1, yielding a ubiquitin-E1 thioester & free AMP. Essential for formation of radiation-induced foci, timely DNA repair & for response to replication stress. Promotes the recruitment of TP53BP1 & BRCA1 at DNA damage sites. |
| TMPO | P42167 | Thymopoietin | Resides in the nucleus. Plays an important role, with LMNA, in the nuclear anchorage of RB1. |
| DSC1 | Q08554 | Desmocollin 1 | Component of intercellular desmosome junctions. Involved in the interaction of plaque proteins & intermediate filaments mediating cell-cell adhesion. |
| PNP | P00491 | Purine Nucleoside Phosphorylase | An enzyme that reversibly catalyzes the phosphorolysis of purine nucleosides. Catalyzes the phosphorolytic breakdown of the N-glycosidic bond in the beta-(deoxy)ribonucleoside molecules, with the formation of the corresponding free purine bases & pentose-1-phosphate. |
| TMED10 | P49755 | Transmembrane P24 Trafficking Protein 10 | Member of the EMP24/GP25L/p24 family, encodes a protein with a GOLD domain. Type I membrane protein that localizes to the plasma membrane & golgi cisternae. Involved in vesicular protein trafficking. Mainly functions in the early secretory pathway. In COPI vesicle-mediated retrograde transport, involved in the biogenesis of COPI vesicles & vesicle coat recruitment. On Golgi membranes, acts as primary receptor for ARF1-GDP, which is involved in COPI-vesicle formation. Increases coatomer-dependent GTPase-activating activity of ARFGAP2. Member of a heteromeric secretase complex & regulates the complex's gamma-secretase activity without affecting its epsilon-secretase activity. Involved in trafficking of G protein-coupled receptors. Regulates F2LR1, OPRM1 & P2RY4 exocytic trafficking from the Golgi to the plasma membrane, contributing to receptor resensitization. Involved in trafficking of amyloid beta A4 protein & soluble APP-beta release (independent of modulation of gamma-secretase activity). As part of the presenilin-dependent gamma-secretase complex, regulates gamma-cleavages of the amyloid beta A4 protein to yield amyloid-beta 40. Involved in organization of the Golgi apparatus. |
| DSP | P15924 | Desmoplakin | Anchors intermediate filaments to desmosomal plaques & forms an obligate component of functional desmosomes. Involved in organization of desmosomal cadherin-plakoglobin complexes into discrete plasma membrane domains. |
| PSMB9 | P28065 | Proteasome Subunit Beta 9 | Subunit is involved in antigen processing to generate class I binding peptides. |
| CNDP2 | Q96KP4 | CNDP Dipeptidase 2 (Metallopeptidase M20 Family) | CNDP2 (tissue carnosinase or peptidase A) is a nonspecific dipeptidase rather than a selective carnosinase. Hydrolyzes a variety of dipeptides including L-carnosine, has a strong preference for Cys-Gly. A functional tumor suppressor in gastric cancer via activation of the mitogen-activated protein kinase (MAPK) pathway. Elevated levels of CNDP2 activates the p38 & JNK MAPK pathways to induce apoptosis. Lower levels of CNDP2 activates the ERK MAPK pathway to promote cell proliferation. Catalyzes the production of N-lactoyl-amino acids from lactate & amino acids by reverse proteolysis. |
| CS | O75390 | Citrate Synthase | A Krebs/tricarboxylic acid cycle enzyme that catalyzes the synthesis of citrate from oxaloacetate and acetyl coenzyme A. Found in nearly all cells capable of oxidative metablism. The protein is nuclear encoded and transported into the mitochondrial matrix, where the mature form is found. |
| C1QBP | Q07021 | Complement C1q Binding Protein | Associates with C1r & C1s to yield the first component of the serum complement system. Known to bind to the globular heads of C1q molecules & inhibit C1 activation. It is the p32 subunit of pre-mRNA splicing factor SF2, as well as a hyaluronic acid-binding protein. |

# **Table S7.** Tau interactome unique to AD− group

| **Gene Symbol** | **UniProtKB** | **Gene Name** | **Function (GeneCards)** |
| --- | --- | --- | --- |
| ERH | P84090 | Enhancer of Rudimentary homolog (Drosophila) | No information |
| H2AFZ | P0C0S5 | H2A Histone Family Member Z | Variant histone H2A replaces conventional H2A in a subset of nucleosomes. Encodes a replication-independent member of the histone H2A family distinct from other members of the family. Studies in mice showed that this particular histone is required for embryonic development & indicate that lack of functional histone H2A leads to embryonic lethality. |
| CALML5 | Q9NZT1 | Calmodulin Like 5 | A novel calcium binding protein expressed in the epidermis and related to the calmodulin family of calcium binding proteins. Functional studies with recombinant protein demonstrate binds calcium and undergoes a conformational change when bound. Abundant expression is detected only in reconstructed epidermis and is differentiating keratinocytes. |
| SNRPD1 | P62314 | Small Nuclear Ribonucleoprotein D1 Polypeptide | Core component of the spliceosomal U1, U2, U4 & U5 small nuclear ribonucleoproteins (snRNPs). |
| H2AFX | P16104 | H2A Histone Family Member X | Variant histone H2A replaces conventional H2A in a subset of nucleosomes. Required for checkpoint-mediated arrest of cell cycle progression in response to low doses of ionizing radiation & for efficient repair of DNA double strand breaks (DSBs) specifically when modified by C-terminal phosphorylation. A replication-independent histone. |
| IGHM | P01871 | Immunoglobulin Heavy Constant Mu | Encodes the C region of the mu heavy chain, which defines the IgM isotype. Naive B cells express the transmembrane forms of IgM & IgD (see IGHD; MIM 1471770) on their surface. During antibody response, activated B cells can switch to the expression of individual downstream heavy chain C region genes by isotype switching. Secreted Ig forms that act as antibodies can be produced by alternative RNA processing of the heavy chain C region sequences. Although the membrane forms of all Ig isotypes are monomeric, secreted IgM forms pentamers, & occasionally hexamers, in plasma |
| RPL11 | P62913 | Ribosomal Protein L11 | Belongs to the L5P family of ribosomal proteins. Located in the cytoplasm. Required for rRNA maturation & formation of the 60S ribosomal subunits. Promotes nucleolar location of PML. |
| IGKC | P01834 | Immunoglobulin Kappa Constant | No information |
| TMSB4X | P62328 | Thymosin Beta 4, X-Linked | Actin sequestering protein that regulates actin polymerization. Involved in cell proliferation, migration, & differentiation. This gene escapes X inactivation & has a homolog on chromosome Y. Binds to & sequesters actin monomers (G actin), thereby inhibiting actin polymerization. |
| FABP5 | Q01469 | Fatty Acid Binding Protein 5 | High specificity for fatty acids. Highest affinity for C18 chain length. Decreasing chain length or introducing double bonds reduces affinity. |
| HLA-DQA2 | P01906 | Major Histocompatibility Complex, Class II, DQ Alpha 2 | Belongs to the HLA class II alpha chain family. Located in intracellular vesicles & plays a central role in the peptide loading of MHC class II molecules by helping to release the CLIP molecule from the peptide binding site. Class II molecules are expressed in antigen presenting cells (B lymphocytes, dendritic cells, macrophages) & are used to present antigenic peptides on the cell surface to be recognized by CD4 T-cells. Binds peptides derived from antigens that access the endocytic route of antigen presenting cells & presents them on the cell surface for recognition by the CD4 T-cells. |
| IFITM1 | P13164 | Interferon Induced Transmembrane Protein 1 | IFN-induced antiviral protein that inhibits the entry of viruses to the host cell cytoplasm, permitting endocytosis, but preventing subsequent viral fusion & release of viral contents into the cytosol. Active against multiple viruses. Plays a key role in the antiproliferative action of IFN-gamma either by inhibiting the ERK activation or by arresting cell growth in G1 phase in a p53-dependent manner. Positive regulator of osteoblast differentiation. |
| SERPINB4 | P48594 | Serpin Family B Member 4 | No information |
| SERPINB3 | P29508 | Serpin Family B Member 3 | Inhibitor of UV-induced apoptosis via suppression of the activity of c-Jun NH(2)-terminal kinase (JNK1). |
| HMGA1 | P17096 | High Mobility Group AT-Hook 1 | Chromatin-associated protein involved in regulation of gene transcription, integration of retroviruses into chromosomes & metastatic progression of cancer cells. Preferentially binds to the minor groove of AT-rich regions in double-stranded DNA. |
| S100A7 | P31151 | S100 Calcium Binding Protein A7 | Member of the S100 family of proteins containing 2 EF-hand calcium-binding motifs. S100 proteins are localized in the cytoplasm and/or nucleus of a wide range of cells. Involved in the regulation of a number of cellular processes such as cell cycle progression and differentiation. The protein is overexpressed in hyperproliferative skin diseases, exhibits antimicrobial activities against bacteria and induces immunomodulatory activities. |
| RPLP1 | P05386 | Ribosomal Protein Lateral Stalk Subunit P1 | Ribosomal phosphoprotein component of the 60S subunit. Belongs to the L12P family of ribosomal proteins. Plays an important role in the elongation step of protein synthesis. Unlike most ribosomal proteins, which are basic, it is acidic. Its C-terminal end is nearly identical to the C-terminal ends of the ribosomal phosphoproteins P0 and P2. Located in the cytoplasm. |
| HSPB1 | P04792 | Heat Shock Protein Family B (Small) Member 1 | Induced by environmental stress & developmental changes. The protein is involved in stress resistance & actin organization. Translocates from the cytoplasm to the nucleus upon stress induction. |
| VIM | P08670 | Vimentin | Class-III intermediate filaments found in non-epithelial cells. Attached to the nucleus, endoplasmic reticulum, and mitochondria, either laterally or terminally. Responsible for maintaining cell shape, integrity of cytoplasm & stabilizing cytoskeletal interactions. Functions as an organizer of critical proteins involved in attachment, migration, and cell signaling. Involved with LARP6 in the stabilization of type I collagen mRNAs for CO1A1 & CO1A2. Involved in immune response & controls the transport of low-density lipoprotein (LDL)-derived cholesterol from lysosomes to the site of esterification. |
| CSTB | P04080 | Cystatin B | A stefin that functions as an intracellular thiol protease inhibitor. Inhibits papain and cathepsins l, h & b. |
| RPS27 | P42677 | Ribosomal Protein S27 | Belongs to the S27E family of ribosomal proteins. Contains a C4-type zinc finger domain that can bind to zinc. Able to bind to nucleic acid. Located in the cytoplasm as a ribosomal component but has been detected in the nucleus. Studies in rat indicate that ribosomal protein S27 is located near ribosomal protein S18 in the 40S subunit & is covalently linked to translation initiation factor eIF3 |
| ISG15 | P05161 | ISG15 Ubiquitin-Like Modifier | A ubiquitin-like protein that is conjugated to intracellular target proteins upon activation by interferon-alpha & interferon-beta. Has chemotactic activity towards neutrophils, direction of ligated target proteins to intermediate filaments, cell-to-cell signaling, & antiviral activity during viral infections. Conjugates of this protein are noncovalently attached to intermediate filaments, and this protein is sometimes secreted. |
| TPT1 | P13693 | Tumor Protein, Translationally-Controlled 1 | Involved in calcium binding and microtubule stabilization. |
| HLA-DPB1 | P04440 | Major Histocompatibility Complex, Class II, DP Beta 1 | HLA-DPB belongs to the HLA class II beta chain paralogues. It plays a central role in the immune system by presenting peptides derived from extracellular proteins. Class II molecules are expressed in antigen presenting cells (APC: B lymphocytes, dendritic cells, macrophages) |
| SRSF7 | Q16629 | Serine And Arginine Rich Splicing Factor 7 | Required for pre-mRNA splicing. Represses the splicing of MAPT/Tau exon 10. |
| GFPT1 | Q06210 | Glutamine--Fructose-6-Phosphate Transaminase 1 | Encodes the first and rate-limiting enzyme of the hexosamine pathway and controls the flux of glucose into the hexosamine pathway. The product of this gene catalyzes the formation of glucosamine 6-phosphate. Regulates the circadian expression of clock genes ARNTL/BMAL1 and CRY1. |
| ABRACL | Q9P1F3 | ABRA C-Terminal Like | No information |
| HLA-DQB1 | P01920 | Major Histocompatibility Complex, Class II, DQ Beta 1 | HLA-DQB1 belongs to the HLA class II beta chain paralogs. This class II molecule is a heterodimer consisting of an alpha (DQA) and a beta chain (DQB), both anchored in the membrane. It plays a central role in the immune system by presenting peptides derived from extracellular proteins. Class II molecules are expressed in antigen presenting cells (APC: B lymphocytes, dendritic cells, macrophages) |
| USMG5 | Q96IX5 | Up-Regulated During Skeletal Muscle Growth 5 Homolog (Mouse) | Plays a critical role in maintaining the ATP synthase population in mitochondria. |
| BTF3 | P20290 | Basic Transcription Factor 3 | Forms a stable complex with RNA polymerase IIB. Required for transcriptional initiation. When associated with NACA, prevents inappropriate targeting of non-secretory polypeptides to the endoplasmic reticulum (ER). Binds to nascent polypeptide chains as they emerge from the ribosome & blocks their interaction with the signal recognition particle, which normally targets nascent secretory peptides to the ER. A transcription factor that can form a stable complex with RNA polymerase II. Required for the initiation of transcription. |
| B2M | P61769 | Beta-2-Microglobulin | Component of the class I major histocompatibility complex (MHC) heavy chain on the surface of nearly all nucleated cells. Involved in the presentation of peptide antigens to the immune system. Has a predominantly beta-pleated sheet structure that can form amyloid fibrils in some pathological conditions. The encoded antimicrobial protein displays antibacterial activity in amniotic fluid. |
| RPL35A | P18077 | Ribosomal Protein L35a | Belongs to the L35AE family of ribosomal proteins. Located in the cytoplasm. The rat protein binds to both initiator & elongator tRNAs. Located at the P site, or P & A sites of the ribosome. Required for proliferation & viability of hematopoietic cells. Plays a role in 60S ribosomal subunit formation. |
| SNRPA | P09012 | Small Nuclear Ribonucleoprotein Polypeptide A | Associates with stem loop II of the U1 small nuclear ribonucleoprotein, which binds the 5' splice site of precursor mRNAs & is required for splicing. The protein autoregulates itself by polyadenylation inhibition of its own pre-mRNA via dimerization & has been implicated in the coupling of splicing & polyadenylation. |
| UBE2L3 | P68036 | Ubiquitin Conjugating Enzyme E2 L3 | Ubiquitin-conjugating enzyme E2 that specifically acts with HECT-type & RBR family E3 ubiquitin-protein ligases. Has activity with the RBR family E3 enzymes, & function like RING-HECT hybrids. Accepts ubiquitin from the E1 complex & catalyzes its covalent attachment to other proteins. Involved in selective degradation of short-lived & abnormal proteins. Down-regulated during the S-phase; involved in progression through the cell cycle. |
| NHP2 | Q9NX24 | NHP2 Ribonucleoprotein | Part of the H/ACA small nucleolar ribonucleoprotein (H/ACA snoRNP) complex, which catalyzes pseudouridylation of rRNA. snoRNPs proteins localize to the dense fibrillar components of nucleoli and to coiled (Cajal) bodies in the nucleus. Both 18S rRNA production and rRNA pseudouridylation are impaired if any one of the four snoRNPs is depleted. The 4 H/ACA snoRNP proteins are components of the telomerase complex. Required for ribosome biogenesis and telomere maintenance. This involves the isomerization of uridine such that the ribose is subsequently attached to C5, instead of the normal N1. |
| CSTA | P01040 | Cystatin A | A stefin that functions as a cysteine protease inhibitor, forming tight complexes with papain & cathepsins B, H, and L. One of the precursor proteins of cornified cell envelope in keratinocytes. Plays a role in epidermal development & maintenance. An intracellular thiol proteinase inhibitor. Has an important role in desmosome-mediated cell-cell adhesion in the lower levels of the epidermis. |
| TPM4 | P67936 | Tropomyosin 4 | Binds to actin filaments in muscle & non-muscle cells. Plays a central role, with the troponin complex, in the calcium dependent regulation of striated muscle contraction. In non-muscle cells is implicated in stabilizing cytoskeleton actin filaments. Binds calcium. |
| RAB11B | Q15907 | RAB11B, Member RAS Oncogene Family | Required for melanosome transport and release from melanocytes. Also regulates V-ATPase intracellular transport in response to extracellular acidosis. |
| PCMT1 | P22061 | Protein-L-Isoaspartate (D-Aspartate) O-Methyltransferase | This enzyme plays a role in protein repair by recognizing and converting D-aspartyl & L-isoaspartyl residues resulting from spontaneous deamidation back to the normal L-aspartyl form. The encoded protein may play a protective role in the pathogenesis of Alzheimer's disease. Plays a role in the repair and/or degradation of damaged proteins. |
| ARG1 | P05089 | Arginase 1 | Involved in step 1 of the subpathway that synthesizes L-ornithine and urea from L-arginine. The type I isoform is a cytosolic enzyme & is expressed predominantly in the liver as a component of the urea cycle. |
| KRT33B | Q14525 | Keratin 33B | Type I hair keratin. Acidic protein that heterodimerizes with type II keratins to form hair and nails. |
| RHOA | P61586 | Ras Homolog Family Member A | Regulates a signal transduction pathway linking plasma membrane receptors to the assembly of focal adhesions & actin stress fibers. Involved in a microtubule-dependent signal required for myosin contractile ring formation during cell cycle cytokinesis. Plays an essential role in cleavage furrow formation. Required for apical junction formation of keratinocyte cell-cell adhesion. Stimulates PKN2 kinase activity. Activated by ARHGEF2, which promotes the exchange of GDP for GTP. Essential for the SPATA13-mediated regulation of cell migration & adhesion assembly & disassembly. The MEMO1-RHOA-DIAPH1 signaling pathway plays an important role in ERBB2-dependent stabilization of microtubules at the cell cortex. Controls the localization of APC & CLASP2 to the cell membrane. Promotes KCNA2 endocytosis. |
| LTF | P02788 | Lactotransferrin | A major iron-binding protein in milk & body secretions with antimicrobial activity. An important component of the non-specific immune system. Demonstrates a broad spectrum of properties, including regulation of iron homeostasis, host defense against a broad range of microbial infections, anti-inflammatory activity, regulation of cellular growth & differentiation & protection against cancer development & metastasis. |
| RAB8B | Q92930 | RAB8B, Member RAS Oncogene Family | A low molecular mass monomeric GTPase that localizes on the cytoplasmic surfaces of distinct membrane-bound organelles. RAB proteins function in intracellular vesicle transport by aiding in the docking and/or fusion of vesicles with their target membranes |
| EIF1AX | P47813 | Eukaryotic Translation Initiation Factor 1A, X-Linked | An essential eukaryotic translation initiation factor. The protein is required for the binding of the 43S complex to the 5' end of capped RNA. Enhances ribosome dissociation into subunits and stabilizes the binding of the initiator Met-tRNA(I) to 40 S ribosomal subunits. |
| RBBP4 | Q09028 | RB Binding Protein 4, Chromatin Remodeling Factor | A ubiquitously expressed nuclear protein that belongs to a highly conserved subfamily of WD-repeat proteins. Component of several complexes that regulate chromatin metabolism, including chromatin assembly factor 1 complex, core histone deacetylase complex, nucleosome remodeling & histone deacetylase complex, PRC2/EED-EZH2 complex, & the nucleosome remodeling factor complex. |
| RAB5B | P61020 | RAB5B, Member RAS Oncogene Family | Involved in protein transport |
| LSP1 | P33241 | Lymphocyte-Specific Protein 1 | Encodes an intracellular F-actin binding protein. Expressed in lymphocytes, neutrophils, macrophages, and endothelium. |
| MT-CO2 | P00403 | Mitochondrially Encoded Cytochrome C Oxidase II | Cytochrome c oxidase is the component of the respiratory chain that catalyzes the reduction of oxygen to water. Subunits 1-3 form the functional core of the enzyme complex. Subunit 2 transfers the electrons from cytochrome c via its binuclear copper A center to the bimetallic center of the catalytic subunit 1. |
| RAB14 | P61106 | RAB14, Member RAS Oncogene Family | Involved in membrane trafficking between the Golgi complex and endosomes during early embryonic development. Regulates the Golgi to endosome transport of FGFR-containing vesicles during early development, key for developing basement membrane & epiblast & primitive endoderm lineages during early postimplantation development. Regulates, together with guanine nucleotide exchange factor DENND6A, the specific endocytic transport of ADAM10, N-cadherin/CDH2 shedding & cell-cell adhesion. |

# **Table S8.** Ontologies of Tau interactome common to AD+ and AD− groups

| Category | Term | Count | % | p-Value | Benjamini | Proteins |
| --- | --- | --- | --- | --- | --- | --- |
| UP_KEYWORDS | Acetylation AD- | 29 | 58% | 1.40E-10 | 2.00E-08 | ABRACL CALML5 CSTA CSTB ERH FABP5 H2AFX H2AFZ HSPB1 HMGA1 LSP1 PCMT1 RPL11 RPL35A RPLP1 RAB11B RAB14 RAB5B RBBP4 S100A7 SRSF7 SERPINB3 SERPINB4 SNRPA TMSB4X TPM4 UBE2L3 USMG5 VIM |
|  | Acetylation AD+ | 41 | 82% | 2.30E-23 | 3.20E-21 | GBE1 CNDP2 EHD1 FKBP1A WDR1 XRCC5 ACAT2 APRT ANXA4 ANXA7 CANX CCT4 CCT7 CS C1QBP ECH1 ESD FH GLUD1 GOT2 GSTO1 HSD17B10 PGD PARP1 PSMA1 PSMB1 PSMB9 PNP RPN1 RPL36 RPS6 SEPT11 SEPT2 SEPT7 SEPT8 SLC25A3 STIP1 TMPO UBE2I UBA1 VDAC2 |
| UP_KEYWORDS | Methylation AD- | 10 | 20% | 5.20E-04 | 1.80E-02 | BTF3 H2AFZ HSPB1 HMGA1 RHOA RAB11B RAB14 RAB8B SNRPD1 SNRPA |
|  | Methylation AD+ | 9 | 18% | 2.30E-03 | 3.20E-02 | RAB8A CCT4 CCT7 CS DSP GOT2 SLC25A3 TMPO TMED10 |
| GOTERM_CC_DIRECT | extracellular exosome AD- | 34 | 68% | 1.30E-16 | 1.70E-14 | A1 B2M CALML5 CSTA CSTB COX2 FABP5 GFPT1 H2AFX H2AFZ HSPB1 IGHM IGKC KRT33B LTF LSP1 PCMT1 RAB11B RAB14 RAB5B RAB8B RHOA RPL11 RPL35A RPLP1 S100A7 SRSF7 SERPINB3 SERPINB4 TPM4 TPT1 UBE2L3 USMG5 VIM |
|  | extracellular exosome AD+ | 36 | 72% | 2.10E-18 | 3.30E-16 | GBE1 CNDP2 EHD1 FKBP1A RAB8A WDR1 ACAT2 APRT ANXA4 ANXA7 CANX CCT4 CCT7 CS DSC1 DSP ERP29 ECH1 ESD FH GOT2 GSTO1 IGHG1 PGD PSMD8 PSMA1 PSMB1 PSMB9 PNP SEPT11 SEPT2 SEPT7 SLC25A3 TMED10 UBA1 VDAC2 |
| GOTERM_MF_DIRECT | poly(A) RNA binding AD- | 15 | 30% | 1.40E-06 | 1.60E-04 | BTF3 CSTB ERH HSPB1 LOC107984923 NHP2 RPL11 RPL35A RPS27 SRSF7 SNRPD1 SNRPA TMSB4X TPT1 UBE2L3 |
|  | poly(A) RNA binding AD+ | 16 | 32% | 2.90E-07 | 4.40E-05 | SLIRP XRCC5 ANXA7 CANX CCT4 CS DSP GOT2 HSD17B10 PARP1 RPN1 RPL36 RPS6 STIP1 UBE2I UBA1 |

# **Table S9.** Ontologies of Tau interactome enriched in AD+ group

| Category | Term | Count | % | p-Value | Benjamini | Proteins |
| --- | --- | --- | --- | --- | --- | --- |
| GOTERM_CC_DIRECT | myelin sheath | 9 | 18% | 6.60E-09 | 5.10E-07 | CANX EHD1 GOT2 SEPT2 SEPT8 SLC25A3 STIP1 VDAC2 WDR1 |
| GOTERM_CC_DIRECT | cytosol | 25 | 50% | 8.80E-07 | 4.60E-05 | APRT ANXA7 CCT4 CCT7 C1QBP CNDP2 FH FKBP1A GBE1 GSTO1 PGD PSMD8 PSMA1 PSMB1 PSMB9 PNP RAB8A RPL18 RPL36 RPS6 SEPT7 UBE2I UBA1 WDR1 XRCC5 |
| UP_KEYWORDS | Phosphoprotein | 37 | 74% | 2.10E-06 | 1.50E-04 | CNDP2 RAB8A SLIRP WDR1 XRCC5 APRT ANXA4 CANX CCT4 C1QBP DSC1 DSP ERP29 ECH1 EHD1 FH GBE1 GLUD1 GOT2 GSTO1 PGD PARP1 PSMD8 PSMA1 PSMB1 PNP RPL18 RPS6 SEPT11 SEPT2 SEPT7 SEPT8 SLC25A3 STIP1 TMPO UBE2I UBA1 |
| UP_KEYWORDS | Cytoplasm | 27 | 54% | 6.60E-06 | 3.00E-04 | CNDP2 FKBP1A RAB8A WDR1 ACAT2 APRT CCT4 CCT7 C1QBP DSP ESD FH GSTO1 PGD PSMA1 PSMB1 PSMB9 PNP RPL18 SEPT11 SEPT2 SEPT7 SEPT8 STIP1 TMPO UBE2I UBA1 |
| KEGG_PATHWAY | Carbon metabolism | 7 | 14% | 2.20E-05 | 1.60E-03 | ACAT2 CS ESD FH GLUD1 GOT2 PGD |
| GOTERM_CC_DIRECT | cytoplasm | 29 | 58% | 2.80E-05 | 1.10E-03 | CNDP FKBP1A WDR1 ACAT2 APRT ANXA4 CCT4 CCT7 C1QBP DSP ESD FH GLUD1 GSTO1 HSD17B10 PSMA1 PSMB1 PSMB9 PNP RPL18 RPL36 RPS6 SEPT11 SEPT2 SEPT7 SEPT8 TMPO UBE2I UBA1 |
| GOTERM_CC_DIRECT | mitochondrion | 14 | 28% | 3.50E-05 | 1.10E-03 | SLIRP ACAT2 CCT7 CS C1QBP ECH1 FH GLUD1 GOT2 HSD17B10 PARP1 SLC25A3 UBA1 VDAC2 |
| GOTERM_CC_DIRECT | nucleolus | 11 | 22% | 7.80E-05 | 2.00E-03 | ACAT2 C1QBP PARP1 RAB8A RPL18 RPL36 RPS6 SEPT2 SEPT7 SLC25A3 XRCC5 |
| GOTERM_CC_DIRECT | melanosome | 5 | 10% | 1.60E-04 | 3.40E-03 | CANX CCT4 ERP29 RPN1 TMED10 |
| GAD_DISEASE | Acquired Immunodeficiency Syndrome\|Disease Progression | 11 | 22% | 1.70E-04 | 3.10E-02 | ACAT2 CCT7 CS C1QBP ECH1 FH GLUD1 GOT2 SLC25A3 SLIRP VDAC2 |
| UP_KEYWORDS | Transit peptide | 8 | 16% | 2.60E-04 | 7.20E-03 | SLIRP CS C1QBP ECH1 FH GLUD1 GOT2 SLC25A3 |
| UP_KEYWORDS | Mitochondrion | 11 | 22% | 2.60E-04 | 8.80E-03 | SLIRP CS C1QBP ECH1 FH GLUD1 GOT2 HSD17B10 SLC25A3 UBA1 VDAC2 |
| UP_KEYWORDS | Proteasome | 4 | 8% | 2.70E-04 | 6.30E-03 | PSMD8 PSMA1 PSMB1 PSMB9 |
| GOTERM_CC_DIRECT | membrane | 16 | 32% | 4.60E-04 | 8.80E-03 | ANXA7 CANX C1QBP DSC1 ERP29 ECH1 EHD1 FKBP1A PARP1 RPN1 RPL18 RPL36 RPS6 SLC25A3 TMPO XRCC5 |
| GOTERM_CC_DIRECT | proteasome complex | 4 | 8% | 5.60E-04 | 9.60E-03 | PSMD8 PSMA1 PSMB1 PSMB9 |
| UP_KEYWORDS | Nucleotide-binding | 13 | 26% | 7.90E-04 | 1.50E-02 | CCT4 CCT7 EHD1 GLUD1 RAB8A SEPT11 SEPT2 SEPT7 SEPT8 UBE2I UBA1 VDAC2 XRCC5 |
| UP_KEYWORDS | Threonine protease | 3 | 6% | 1.00E-03 | 1.80E-02 | PSMA1 PSMB1 PSMB9 |
| UP_KEYWORDS | GTP-binding | 6 | 12% | 1.30E-03 | 2.00E-02 | GLUD1 RAB8A SEPT11 SEPT2 SEPT7 SEPT8 |
| GOTERM_CC_DIRECT | nucleus | 26 | 52% | 1.40E-03 | 2.20E-02 | RAB8A SLIRP XRCC5 ACAT2 ANXA4 ANXA7 CS C1QBP DSP PGD PARP1 PSMD8 PSMA1 PSMB1 PSMB9 PNP RPL18 RPS6 SEPT2 SEPT7 SLC25A3 STIP1 TMPO UBE2I UBA1 VDAC2 |
| GOTERM_CC_DIRECT | proteasome core complex | 3 | 6% | 1.40E-03 | 2.00E-02 | PSMA1 PSMB1 PSMB9 |
| GOTERM_CC_DIRECT | nonmotile primary cilium | 3 | 6% | 1.70E-03 | 2.20E-02 | RAB8A SEPT2 SEPT7 |
| GOTERM_CC_DIRECT | mitochondrial matrix | 6 | 12% | 1.80E-03 | 2.10E-02 | CS C1QBP FH GLUD1 GOT2 HSD17B10 |
| GOTERM_CC_DIRECT | desmosome | 3 | 6% | 1.90E-03 | 2.10E-02 | DSC1 DSP UBA1 |
| UP_KEYWORDS | Glycosyltransferase | 5 | 10% | 2.40E-03 | 2.90E-02 | APRT GBE1 PARP1 PNP RPN1 |

# **Table S10.** Ontologies of Tau interactome enriched in AD− group

| Category | Term | Count | % | p-Value | Benjamini | Proteins |
| --- | --- | --- | --- | --- | --- | --- |
| GOTERM_BP_DIRECT | negative regulation of peptidase activity | 4 | 8% | 1.40E-05 | 5.20E-03 | CSTA CSTB ERPINB3 SERPINB4 |
| GOTERM_BP_DIRECT | antigen processing and presentation | 5 | 10% | 1.70E-05 | 3.20E-03 | HLA-DPB1 HLA-DQA2 HLA-DQB1 RAB5B RAB8B |
| GOTERM_CC_DIRECT | focal adhesion | 8 | 16% | 6.80E-05 | 5.30E-03 | B2M HSPB1 HMGA1 RHOA RPLP1 S100A7 TPM4 VIM |
| UP_KEYWORDS | Ribonucleoprotein | 7 | 14% | 7.00E-05 | 4.80E-03 | NHP2 RPL11 RPL35A RPS27 RPLP1 SNRPD1 SNRPA |
| KEGG_PATHWAY | Tuberculosis | 7 | 14% | 1.10E-04 | 1.10E-02 | CALML5 HLA-DPB1 HLA-DQA2 HLA-DQB1 LSP1 RAB5B RHOA |
| UP_KEYWORDS | Immunity | 8 | 16% | 1.70E-04 | 7.70E-03 | B2M HLA-DPB1 HLA-DQA2 HLA-DQB1 IGKC IFITM1 ISG15 LTF |
| GOTERM_BP_DIRECT | retina homeostasis | 4 | 8% | 1.90E-04 | 2.40E-02 | B2M HSPB1 IGKC LTF |
| KEGG_PATHWAY | Systemic lupus erythematosus | 6 | 12% | 2.90E-04 | 1.40E-02 | H2AFX H2AFZ HLA-DPB1 HLA-DQA2 HLA-DQB1 SNRPD1 |
| GOTERM_CC_DIRECT | ER to Golgi transport vesicle membrane | 4 | 8% | 3.50E-04 | 1.80E-02 | B2M HLA-DPB1 HLA-DQA2 HLA-DQB1 |
| GOTERM_MF_DIRECT | GDP binding | 4 | 8% | 4.50E-04 | 2.50E-02 | RAB11B RAB14 RAB5B RAB8B |
| GOTERM_CC_DIRECT | extracellular space | 12 | 24% | 6.10E-04 | 2.30E-02 | A1 B2M CSTA CSTB HSPB1 IGHM IGKC KRT33B LTF SERPINB3 SERPINB4 TPT1 |
| UP_KEYWORDS | Prenylation | 5 | 10% | 6.80E-04 | 1.90E-02 | RAB11B RAB14 RAB5B RAB8B RHOA |
| UP_KEYWORDS | MHC II | 3 | 6% | 7.40E-04 | 1.70E-02 | HLA-DPB1 HLA-DQA2 HLA-DQB1 |
| GOTERM_CC_DIRECT | cytosolic large ribosomal subunit | 4 | 8% | 7.60E-04 | 2.30E-02 | NHP2 RPL11 RPL35A RPLP1 |
| GOTERM_MF_DIRECT | MHC class II receptor activity | 3 | 6% | 7.80E-04 | 2.80E-02 | HLA-DPB1 HLA-DQA2 HLA-DQB1 |
| KEGG_PATHWAY | Amoebiasis | 5 | 10% | 1.20E-03 | 3.90E-02 | A1 HSPB1 RAB5B SERPINB3 SERPINB4 |
| GOTERM_CC_DIRECT | MHC class II protein complex | 3 | 6% | 1.50E-03 | 3.80E-02 | HLA-DPB1 HLA-DQA2 HLA-DQB1 |
